# DNA methylation abnormalities of imprinted genes in congenital heart disease: a pilot study

**DOI:** 10.1186/s12920-020-00848-0

**Published:** 2021-01-06

**Authors:** Shaoyan Chang, Yubo Wang, Yu Xin, Shuangxing Wang, Yi Luo, Li Wang, Hui Zhang, Jia Li

**Affiliations:** 1 Beijing Municipal Key Laboratory of Child Development and Nutriomics, Capital Institute of Pediatrics, Beijing, 100020 China; 2 Department of Cardiac Surgery, Children’s Hospital Affiliated to Capital Institute of Pediatrics, No. 2 Yabao Road, Chao Yang District, Beijing, 100020 China; 3 Clinical Physiology Laboratory, Institute of Pediatrics, Guangzhou Women and Children’s Medical Center, Guangzhou Medical University, No. 9 Jinsui Road, Tianhe District, Guangzhou City, 510000 Guangdong Province China; 4 Guangdong Provincial Key Laboratory of Research in Structural Birth Defect Disease, Guangzhou Women and Children’s Medical Center, Guangzhou Medical University, Guangzhou, 510000 Guangdong Province China

**Keywords:** Congenital heart disease, Imprinted gene, Methylation modification, Germline differential methylation regions

## Abstract

**Background:**

Congenital heart disease (CHD) is resulted from the interaction of genetic aberration and environmental factors. Imprinted genes, which are regulated by epigenetic modifications, are essential for the normal embryonic development. However, the role of imprinted genes in the etiology of CHD remains unclear.

**Methods:**

After the samples were treated with bisulfate salt, imprinted genes methylation were measured by matrix-assisted laser desorption/ionization time-of-flight mass spectrometry. T test and One-way ANOVA were performed to evaluate the differences among groups. Odds ratios (ORs) were performed to evaluate the incidence risk of CHD in relation to methylation levels.

**Results:**

We investigated the alterations of imprinted gene germline differential methylation regions (gDMRs) methylation in patients with CHD. Eighteen imprinted genes that are known to affect early embryonic development were selected and the methylation modification genes were detected by massarray in 27 CHD children and 28 healthy children. Altered gDMR methylation level of 8 imprinted genes was found, including 2 imprinted genes with hypermethylation of *GRB10* and *MEST* and 6 genes with hypomethylation of *PEG10*, *NAP1L5*, *INPP5F*, *PLAGL1*, *NESP* and *MEG3*. Stratified analysis showed that the methylation degree of imprinted genes was different in different types of CHD. Risk analysis showed that 6 imprinted genes, except *MEST* and *NAP1L5,* within a specific methylation level range were the risk factors for CHD

**Conclusion:**

Altered methylation of imprinted genes is associated with CHD and varies in different types of CHD. Further experiments are warranted to identify the methylation characteristics of imprinted genes in different types of CHD and clarify the etiologies of imprinted genes in CHD.

## Background

Congenital heart disease (CHD) is the most common type of congenital malformations. The etiology for CHD is complex, due to the interaction of genetic aberration and environmental factors, profoundly influenced by conditions in fetal life [1]. Despite the diversity of the CHD category, there appear to be shared epigenetic factors in the etiology of CHD for a couple of reasons. First, approximately 500 genes have been related to CHDs, but only accounting for 10% of CHD cases [2]. Second, recent research has shown that gene expression can be altered by chemical modifications to the DNA and associated proteins in the nucleus, the epigenome. These modifications can be altered in response to environmental factors, such as ambient air pollution and maternal cigarette smoking after adjusting for socioeconomic status, etc. [1].

Among the different epigenetic mechanisms, the potential relationship between abnormal DNA methylation and CHD has been increasingly recognized. DNA methylation has been found to be highly dynamic with the feature of demethylation in cardiomyocyte-associated gene sets during cardiomyocyte development [3]. Studies have shown that there are hypermethylation of myocardial-related genes in myocardial tissues of CHD, which is closely related to gene downregulation [4, 5]. In patients with CHD, decreased transcriptional activity of *CITE2, ZIC3, NR2F2* and *BRG1* is associated with abnormal methylation [6–9].

DNA methylation has a major influence on the establishment of imprinting markers [10]. Several human disorders have been found to be associated with varied methylation modifications at imprinting control regions. For example, Silver-Russell syndrome is characterized by hypomethylation and Prader-Willi Syndrome by hypermethylation of the related imprinted genes [11]. Epigenetic imprinting is particularly vulnerable in early embryonic development. Exploring the epigenetic modification of imprinted genes may provide etiologies of congenital disorders including CHD.

In placental mammals, the imprinted genes regulate embryonic development. At present, about 94 human imprinted genes have been identified [12]. It has been proposed that *IGF2* and *GRB10* and co-regulated genes of *PEG1/MEST*, *GTL2/MEG3*, *CDKN1C, PLAGL1* and *DLK1* regulates embryonic growth and development by forming a proposed "imprinted gene network" [13]. The most well-studied example of involving in cardiac development is *DLK1*-*DIO3*, encoded ncRNAs, which participate in the commitment of the mesoderm to different subsets of a specific cardiac cell lineage through many *DLK1*-*DIO3* ncRNAs [14]. However, there has been no study on the epigenetic modification of imprinted genes in CHD.

Therefore, we investigated the alterations of imprinted gene methylation in patients with CHD through methylation modification. We selected 18 imprinted genes that are known to play an important role in early embryonic developments [15].

## Methods

### Sample collection

The study was approved by the Ethics Committee of the Capital Institute of Pediatrics. Written informed consent was obtained from parents of the children. These samples were obtained from children with CHD who visited the Capital Institute of Pediatrics during the biennium 2014–2015, and were diagnosed as CHD by echocardiography and other related tests, ranging in age from 1 to 120 months. According to the International Classification Standard of Diseases, the diagnosis of existing cases was strictly classified, and a complete database of CHD research is established. Moreover, the children with CHD were excluded from other congenital and acquired abnormal diseases, such as congenital mental retardation, genetic metabolic diseases, etc. As control group, 28 controls of blood samples were selected from the physical examination children. They were same age and race and had no developmental deformities or other diseases. Peripheral blood samples were collected from 27 children with CHD and 28 healthy children as controls in Capital Institute of Pediatrics (Table 3). In details, 2 ml venous blood was collected into the evacuated tubes without anticoagulant (Becton Dickinson). Blood samples were immediately centrifuged at 2500 rpm for 10 min. The separated plasma was aliquot without reducing agent and stored at − 20 °C.

### DNA extraction

Genomic DNA was extracted from peripheral blood with the Blood and Tissue DNA Kit (65904, QIAGEN, Dusseldorf, Germany) according to manufacturer’s instructions. The concentration and purity of the DNA were determined by nanodrop and agarose gel electrophoresis. DNA with an OD260/280 absorbance ratio of 1.8 was used for subsequent work (Additional files 1–9: Figures S1–S9).

### Bisulfite treatment

A total of 500 ng genomic DNA from each sample underwent bisulfite treatment using the EZ DNA methylation kit (D5001, Zymo Research, Irvine, CA, USA) according to the manufacturer’s instruction. The quality of the bisulfite conversion was controlled by using PCR products that had no methyl group. Sequencing results confirmed that 96.6% of cytosine residues were converted in previous research [16].

### PCR amplification

Eighteen imprinted genes were selected according to WAMZDEX, Gene imprint, Catalogue of Imprinted Genes and other imprinted gene analysis resources (Additional file 10: Table S1). gDMRs sequences of the selected imprinted genes were retrieved to confirm using the UCSC database (Additional file 11: Table S2). The primers were designed for the quantitative analysis of the methylation level with Methprimer (http://epidesigner.com/). An additional T7 promoter tag was added to each reverse primer for in vivo transcription, and a 10-mer tag to the forward primer to balance the melting temperature.

### Sap reaction

The sap reaction was performed to eliminate residual dNTP in PCR products to facilitate mass spectrometry detection. The specific steps were to add 2 ul sap mixture into 5 ul PCR product, and to conduct vortex mixing before centrifugation. The reaction conditions were 37 °C for 20 min and 85 °C for 5 min.

### Methylation analyses

As the previously published method [16], the Sequenom MassARRAY platform (CapitalBio, Beijing, China) was used to perform the quantitative methylation analysis of the imprinted genes. This system uses matrix-assisted laser desorption/ionization time-of-flight (MALDI-TOF) mass spectrometry in combination with RNA base-specific cleavage (MassCLEAVE). A detectable pattern was then analyzed for its methylation status. The spectral methylation ratios were generated using Epityper software version 1.0 (Sequenom, San Diego, CA, USA).

### Statistical analyses

Data are presented as mean and standard deviation. The methylation levels in the gDMRs of imprint genes were compared between CHD and control groups by independent samples t-test. One-way ANOVA was performed to evaluate the differences among different CHD subtypes and control groups. Odds ratios (ORs) was performed to evaluate the incidence risk of CHD in relation to methylation levels. P < 0.05 was considered significant. GraphPad Prism 7 software (GraphPad Software, San Diego, California, USA) was used to visually display the results of analysis. Data were stored in the EPI 3.1 Database (EpiData Association, Odense, Denmark) and analyzed with the SPSS 18.0 software package (McGraw-Hill Inc., New York, NY, USA).

## Results

### Comparison of the altered methylation levels of the imprinted genes between CHD and control groups

**Fig. 1:**
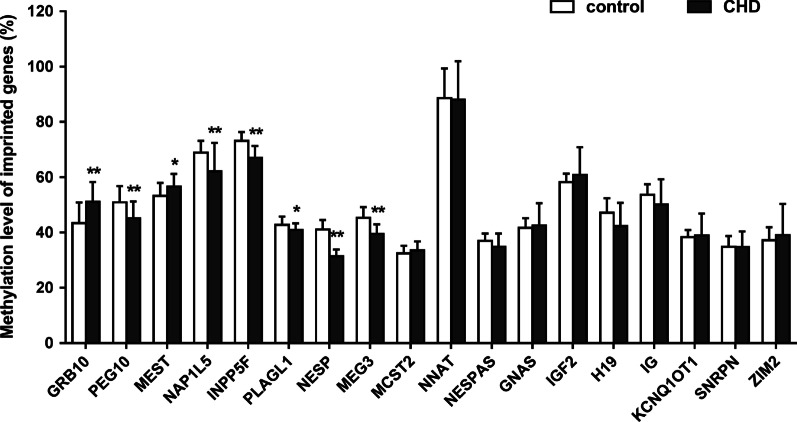
Altered methylation level of 18 imprinted genes between CHD and control groups. Methylation level of 18 imprinted genes between CHD and control groups. Compared with the normal group, 8 of the 18 imprinted genes in CHD showed abnormal methylation modification including *GRB10*, *MEST*, *PEG10*, *NAP1L5*, *INPP5F*, *PLAGL1*, *NESP* and *MEG3*. Among them, the two genes including *GRB10* and *MEST* showed increased methylation, while the others showed decreased methylation. *P < 0.05; **P < 0.01

Table 1 showed the values and statistical results(detailed results including mean methylation and CpG sites methylation of each printed genes studied in Additional file 12: Table S3 and Additional files 13–30: Table S4–S21, respectively). Compared with control group, 8 of the 18 selected genes were found to be significantly different in CHD group; two of them were hypermethylated and 6 hypomethylated. The two hypermethylated-imprinted genes were *GRB10* and *MEST*, in which *GRB10* increased from 43.42% in the normal group to 51.12% (*P* < 0.01) in the CHD group and *MEST* increased from 53.22 to 56.6% (*P* < 0.05). The 6 hypomethylation-imprinted genes were *PEG10*, *NAP1L5*, *INPP5F*, *PLAGL1*, *NESP* and *MEG3*, in which *PEG10* decreased from 50.92% in control to 45.17% in CHD group (*P* < 0.01), *NAP1L5* decreased from 68.86 to 62.12% (*P* < 0.01), *INPP5F* decreased from 73.17 to 67.02% (*P* < 0.01), *PLAGL1* decreased from 42.80 to 40.97%,(*P* < 0.05), *NESP* decreased from 41.12 to 31.31% (*P* < 0.01), and *MEG3* decreased from 45.31 to 39.53%(*P* < 0.01) (Fig. 1; Additional files 1–8: Figures S1–S8).

**Table 1:** Type of CHD and gender distribution in CHD and control groups

Type of CHD		Number of cases		*P* value
		Male	Female	
*CHD*				
Ventricular septal defect		10	7	0.71
Atrial septal defect		6	6	0.40
Valvular malformation		3	4	0.30
Tetralogy of Fallot		4	0	0.15
Patent ductus arteriosus		1	3	0.13
Coarctation of aorta		2	1	0.93
Others		2	1	0.93
Total CHD		17	10	0.92
Controls		18	10	

Others included pulmonary vascular obstruction (n = 2) and congenital diaphragmatic hernia (n = 1); valvular malformation included aortic (n = 4) and mitral (n = 3) valve stenosis or regurgitation

### Altered methylation levels of imprinted genes in CHD classifications

**Fig. 2:**
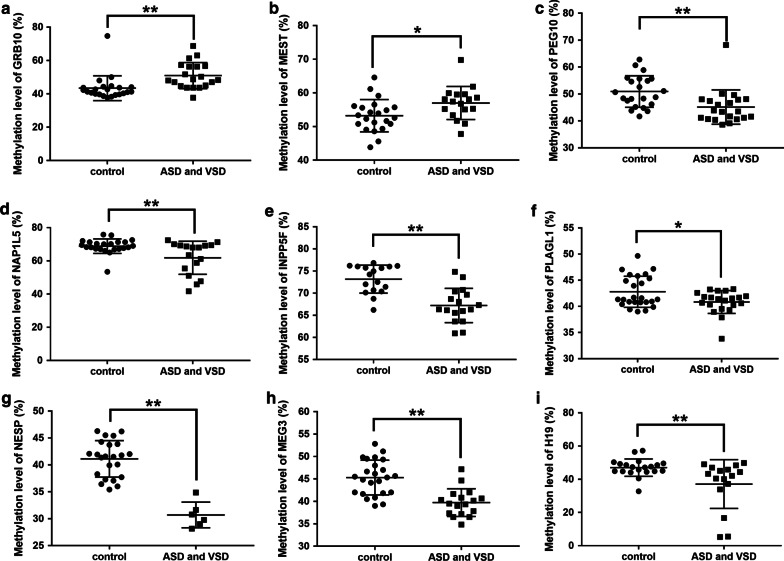
Altered methylation level of the imprinted genes between ASD and VSD and control groups. **a**, **b** Imprinted genes up-regulated by methylation between ASD/VSD and control groups. There were two imprinted genes with increasing methylation including *GRB10* and *MEST* in ASD and VSD groups, compared with the normal group. **c**, **i** Imprinted genes up-regulated by methylation between ASD and VSD and control groups. Compared with the normal group, 7 of the 18 imprinted genes in ASD and VSD showed decreasing methylation modification including *PEG10*, *NAP1L5*, *INPP5F*, *PLAGL1*, *NESP*, *MEG3* and H19. ASD and VSD, atrial and ventricular septal defect. *P < 0.05; **P < 0.01

**Fig. 3:**
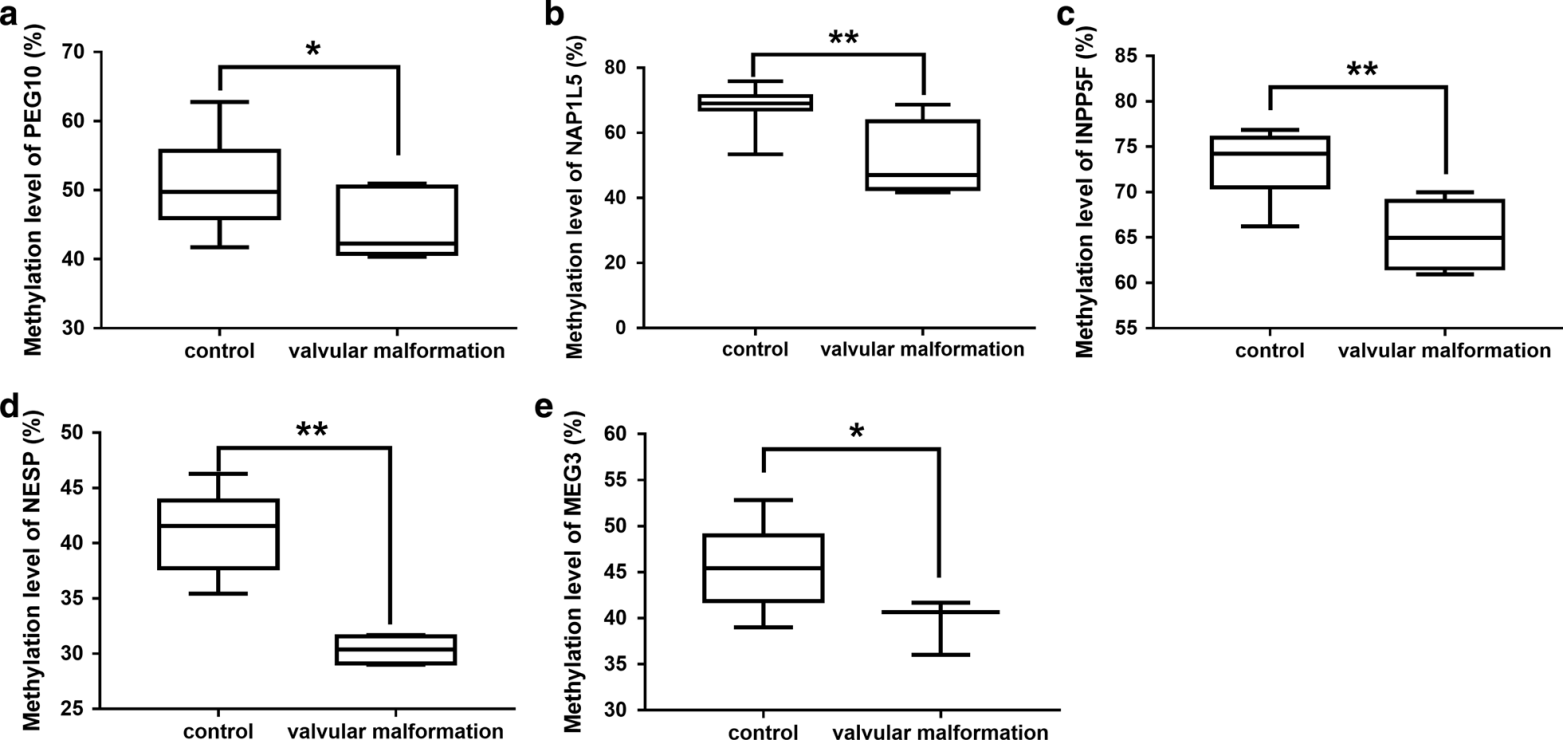
Altered methylation level of the imprinted genes between valvular malformation and control groups. **a**, **e** Hypomethylation of imprinted genes between ASD and VSD and control groups. Compared with the normal group, 5 of the 18 imprinted genes in ASD and VSD showed decreasing methylation modification including *PEG10*, *NAP1L5*, *INPP5F*, *NESP* and *MEG3*. *P < 0.05; **P < 0.01

**Fig. 4:**
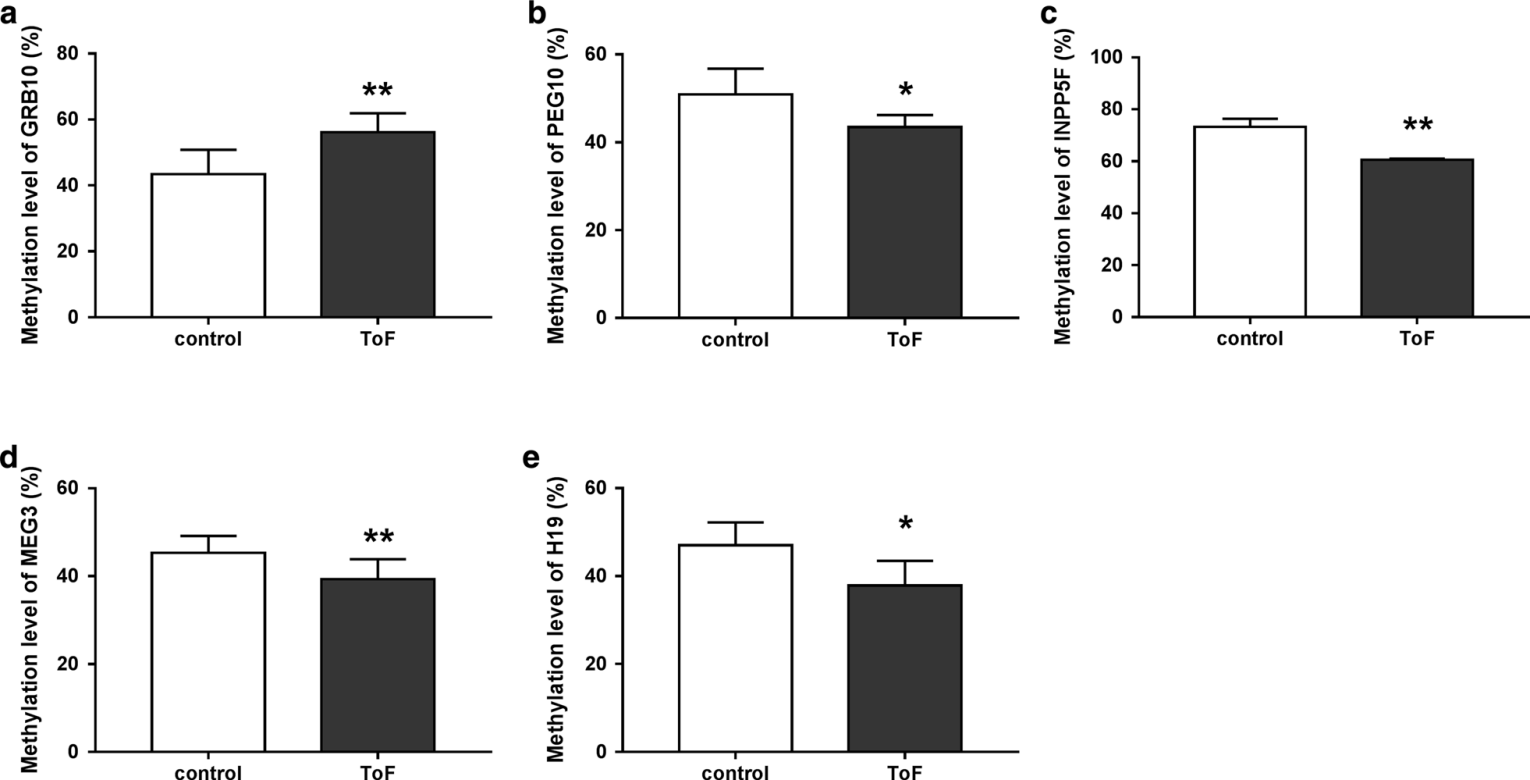
Altered methylation level of the imprinted genes between ToF and control groups. **a** Hypermethylation of GRB10 gene between ToF and control groups. **b**, **e** Hypomethylation of PEG10, INPP5F, MEG3 and H19 respectively between ToF and control groups. Compared with the normal group, 4 of the 18 imprinted genes in ToF showed decreasing methylation modification including PEG10, INPP5*F*, *MEG3* and H19. ToF, Tetralogy of Fallot. *P < 0.05; **P < 0.01

**Fig. 5:**
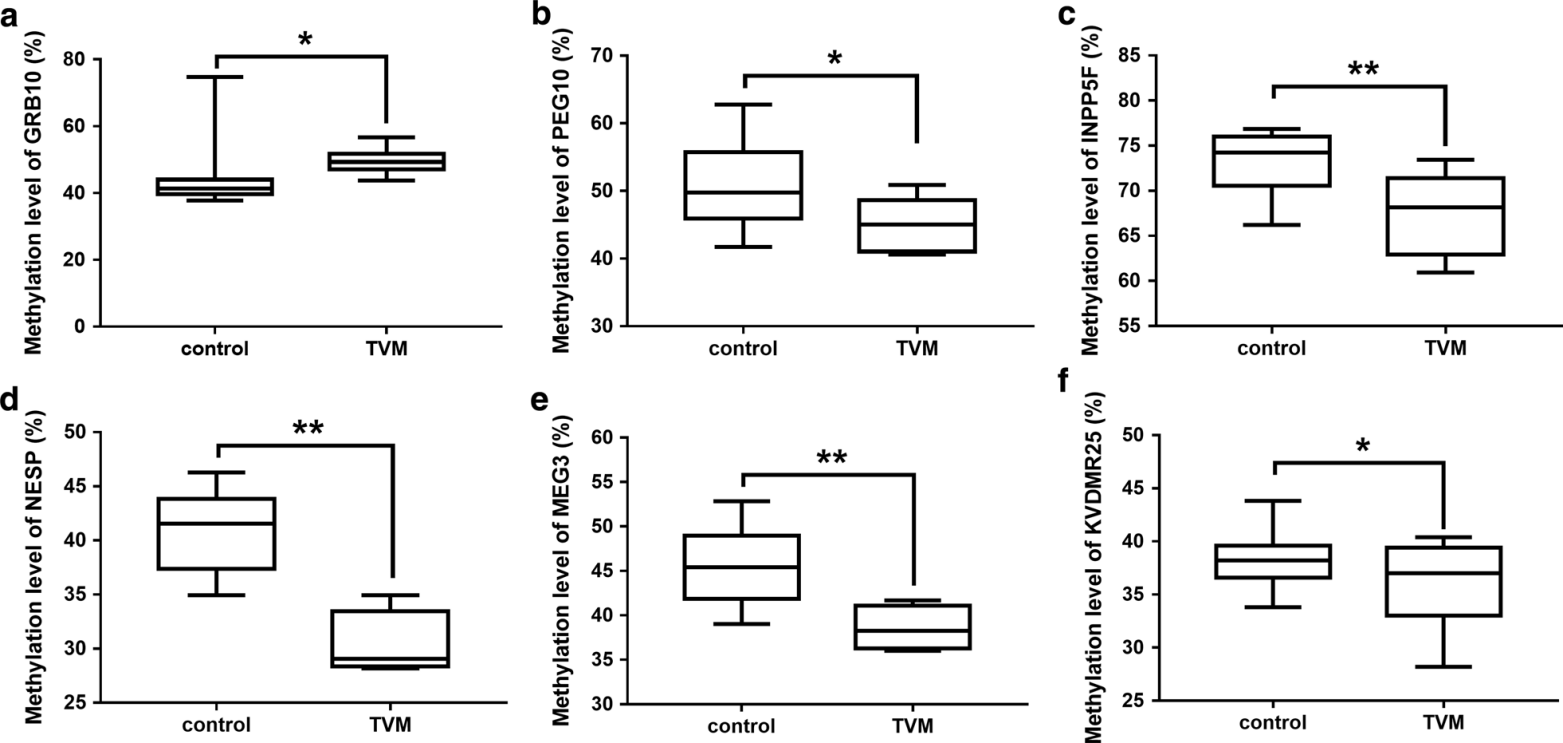
Altered methylation level of the imprinted genes between thoracic vascular malformation and control groups. **a** Hypermethylation of *GRB10* gene between thoracic vascular malformation and control groups. **b**–**f** Hypomethylation of PEG10, INPP5F, NESP, MEG3 and KCNQ1OT1 respectively between thoracic vascular malformation and control groups. Compared with the normal group, 5 of the 18 imprinted genes in thoracic vascular malformation showed decreasing methylation modification including *PEG10*, *INPP5F*, *NESP*, *MEG3* and KCNQ1OT1.TVM, thoracic vascular malformation. *P < 0.05; **P < 0.01

CHD was classified into four groups including atrial and ventricular septal defect (ASD and VSD), valvular malformation (aortic and mitral stenosis or regurgitation), thoracic vascular malformation (coarctation of aorta, patent ductus arteriosus) and Tetralogy of Fallot (ToF). In ASD and VSD patients, 9 genes showed methylation changes, as compared with control group, whereby 2 imprinted genes (the *GRB10* and *MEST*) were hypermethylated (Fig. 2a, b; Additional file 9: Figure S9a) and 7 (*PEG10*, *NAP1L5*, *INPP5F*, *PLAGL1*, *NESP*, *MEG3* and H19) hypomethylation (Fig. 2c–i; Additional file 9: Figure S9a). In valvular malformation group, the methylations of 5 imprinted genes were lower than that of control group, including *PEG10*, *NAP1L5*, *INPP5F*, *NESP* and *MEG3* (Fig. 3; Additional file 9: Figure S9b). In ToF group, methylation of 5 imprinted genes changed, including *GRB10* with higher methylation than that in control group (Fig. 4a; Additional file 9: Figure S9c) and *PEG10*, *INPP5F*, *MEG3* and *H19* with lower methylation (Fig. 4b–e; Additional file 9: Figure S9c). There were differences in methylation of 6 imprinted genes between thoracic vascular malformation and control groups, including hypermethylation of the *GRB10* gene and hypomethylation of *PEG10*, *INPP5F*, *NESP*, *MEG3* and *KCNQ1OT1* respectively (Fig. 5; Additional file 9: Figure S9d). Taken together, 3 imprinted genes (*PEG10*, *INPP5F* and *MEG3*) were hypomethylated in all four CHD classifications. *NESP* hypomethylation occurred in all CHDs except ToF, while *GRB10* hypermethylation occurred in all CHDs except valvular malformation.

## Risk analysis between germline differential methylation regions (gDMRs) methylation levels of imprinted genes and CHD

In order to develop a model for assessing the risk of CHD based on the amplitude of methylation levels of gDMRs of imprinted genes, CHD samples were categorized as Q1-Q4 according to the quartiles of methylation levels in control group. As shown in Table 2, the gDMRs of 8 differentially imprinted genes were further analyzed to examine the relationship between methylation changes of these genes and risk of CHD. Compared to control group, the gDMRs methylation level of *NESP* gene was lower in all CHDs. Methylation modification of two imprinted genes were elevated at 75th percentile level in CHD, with *GRB10* in 21 of 25 patients and *MEST* in 10 of 20 patients. There were 4 imprinted genes whose methylation level of gDMRs were lower at 25th percentile level in CHD including14 of 25 cases of *PEG10*, 17 of 22 cases of *INPP5F*, 11 of 26 cases of *PLAGL1* and 16 of 20 cases of *MEG3* respectively. When logistic regression analysis was used with adjustment to sex and compared with control group, the risk of CHD was 21 times higher than that of control group when *GRB10* methylation was higher than 44%(*P* < 0.05). When the methylation levels of *PEG10*, *INPP5F*, *PLAGL1* and *MEG3* were lower than 45.2%, 70.39%, 40.76% and 41.83%, respectively, the risk of CHD increased by 16.8(*P* < 0.05), 11.06(*P* < 0.01), 11(*P* < 0.05) and 12(*P* < 0.01)times. Due to the small sample size, confidence intervals were wide (Table 3).

**Table 2:** Risk analysis results of DMRs methylation levels of imprinted genes and CHD occurrence

Imprinted genes	Quantile percentages	Controls n (%)	CHD n (%)	Adjusted OR^a^ (95% CI^b^)	Adjusted *P* value
*GRB10*	Q_1_^c^ (< 39.67%)	6(26.1%)	1(4%)	1(reference)	
	Q_2_–Q_3_^d^ (39.67%–44%)	11(47.8%)	3(12%)	1.64(0.14–19.39)	0.696
	Q_4_^e^ (> 44%)	6(26.1%)	21(84%)	21.00(2.10–210.14)	0.01
*MEST*	Q_1_ (< 49.36%)	5(22.7%)	1(5%)	1(reference)	
	Q_2_–Q_3_ (49.36%–56.07%)	12(54.5%)	9(45%)	3.75(0.371–37.95)	0.263
	Q_4_ (> 56.07%)	5(22.7%)	10(50%)	10.00(0.907–110.28)	0.06
*PEG10*	Q_1_ (< 45.2%)	5(22.7%)	14(56%)	16.80(1.60–176.23)	0.019
	Q_2_–Q_3_ (45.2%–55.7%)	11(50%)	10(40%)	5.46(0.56–53.52)	0.145
	Q_4_ (> 55.7%)	6(27.3%)	1(4%)	1(reference)	
*NAP1L5*	Q_1_ (< 67.14%)	6(26.1%)	9(45%)	3.50(0.64–19.20)	0.149
	Q_2_–Q_3_ (67.14%–71.29%)	10(43.5%)	8(40%)	1.87(0.36–9.64)	0.456
	Q_4_ (> 71.29%)	7(30.4%)	3(15%)	1(reference)	
*INPP5F*^f^	Q_1_ (< 70.39%)	4(23.5%)	17(77.3%)	11.06(2.47–49.53)	0.002
	Q_2_–Q4 (≥ 70.39%)	13(76.5%)	5(22.7%)	1(reference)	
*PLAGL1*	Q_1_ (< 40.76%)	6(24%)	11(42.3%)	11.00(1.06–114.09)	0.045
	Q_2_–Q_3_ (40.76%–45.82%)	13(52%)	14(53.8%)	6.46(0.68–61.16)	0.104
	Q_4_ (> 45.82%)	6(24%)	1(3.8%)	1(reference)	
*MEG3*^f^	Q_1_ (< 41.83%)	6(25%)	16(80%)	12.00(2.86–50.31)	0.001
	Q_2_–Q4 (≥ 41.83%)	18(75%)	4(20%)	1(reference)	

^a^Odds ratio

^b^Confidence interval

^c^25th percentile

^d^25th percentile to 75th percentile

^e^75th percentile. Cut-off value was defined as 25th and 75th percentiles of the control group methylation level. Adjusted for sex by logistic regression

^f^Indicates that the maximum value of the imprinting gene cases was less than 75% of control group and could not be calculated, so there were divided into two groups

**Table 3:** Risk analysis results of gDMRs methylation levels of imprinted genes and CHD occurrence

Imprinted genes	Quantile percentages	Controls n (%)	CHD n (%)	Adjusted OR^a^ (95% CI^b^)
*GRB10*	Q_1_^c^ (< 39.67%)	6(26.1%)	1(4%)	1(reference)
	Q_2_–Q_3_^d^ (39.67%–44%)	11(47.8%)	3(12%)	1.64(0.14–19.39)
	Q_4_^e^ (> 44%)	6(26.1%)	21(84%)	21.00(2.10–210.14)
*MEST*	Q_1_ (< 49.36%)	5(22.7%)	1(5%)	1(reference)
	Q_2_–Q_3_ (49.36%–56.07%)	12(54.5%)	9(45%)	3.75(0.371–37.95)
	Q_4_ (> 56.07%)	5(22.7%)	10(50%)	10.00(0.907–110.28)
*PEG10*	Q_1_ (< 45.2%)	5(22.7%)	14(56%)	16.80(1.60–176.23)
	Q_2_–Q_3_ (45.2%–55.7%)	11(50%)	10(40%)	5.46(0.56–53.52)
	Q_4_ (> 55.7%)	6(27.3%)	1(4%)	1(reference)
*NAP1L5*	Q_1_ (< 67.14%)	6(26.1%)	9(45%)	3.50(0.64–19.20)
	Q_2_–Q_3_ (67.14%–71.29%)	10(43.5%)	8(40%)	1.87(0.36–9.64)
	Q_4_ (> 71.29%)	7(30.4%)	3(15%)	1(reference)
*INPP5F*^f^	Q_1_ (< 70.39%)	4(23.5%)	17(77.3%)	11.06(2.47–49.53)
	Q_2_–Q4 (≥ 70.39%)	13(76.5%)	5(22.7%)	1(reference)
*PLAGL1*	Q_1_ (< 40.76%)	6(24%)	11(42.3%)	11.00(1.06–114.09)
	Q_2_–Q_3_ (40.76%–45.82%)	13(52%)	14(53.8%)	6.46(0.68–61.16)
	Q_4_ (> 45.82%)	6(24%)	1(3.8%)	1(reference)
*MEG3*^f^	Q_1_ (< 41.83%)	6(25%)	16(80%)	12.00(2.86–50.31)
	Q_2_–Q4 (≥ 41.83%)	18(75%)	4(20%)	1(reference)

^a^Odds ratio

^b^Confidence interval

^c^25th percentile

^d^25th percentile to 75th percentile

^e^75th percentile. Cut-off value was defined as 25th and 75th percentiles of the control group methylation level. Adjusted for sex by logistic regression

^f^Indicates that the maximum value of the imprinting gene cases was less than 75% of control group and could not be calculated, so there were divided into two groups

## Discussion

It is well known that the epigenetic regulation of imprinted genes is involved in many embryonic developmental processes, including cardiac development [17, 18]. At present, DNA methylation disorder is considered as one of the important factors in the occurrence and development of CHD [4]. However, the specific role of the imprinted gene methylation regulation on cardiac development remains unclear. In this study, we found for the first time the abnormal gDMR methylation changes of 8 imprinted genes (*GRB10*, *MEST*, *PEG10*, *NAP1L5*, *INPP5F*, *PLAGL1*, *NESP* and *MEG3*) in patients with CHD. The methylations of imprinted genes varied with the heterogeneity of CHDs. Six of them were associated with significantly higher risk of CHD except *MEST* and *NAP1L5*.

The etiology of CHD is complex, involving the interaction of environmental and genetic factors, leading to developmental phenotypes that regulate morbidity and severity. The potential relationship between alterations of genomic methylation and CHD is increasingly recognized. The dynamic changes of DNA methylation in cardiomyocyte-related gene sets during cardiac development suggest that DNA methylation modification is essential for the occurrence of cardiac diseases [3, 4]. One study has confirmed that there are significant differences in methylation of genes associated with muscle contraction and cardiomyopathy in CHD [4]. Genomic imprinting is a process of epigenetic modification, and loss of imprinting may lead to abnormal embryonic development [13]. DMR plays an important role in the establishment of gene imprinting by targeting imprinted gene. Deletion or abnormal methylation of DMR might result in the expression disorder of the imprinted gene cluster [19]. Specifically, we selected DMRs in the imprinting control region of imprinted genes studied, i.e., gDMRs, which are established during gametogenesis and play a key role in the imprinting of imprinted gene clusters [19, 20]. The small changes in gDMRs found in our study might be relevant in the etiology of CHD, which was supported by the stratified analysis. The relatively wide confidence interval in the risk analysis was due largely to the small sample size in this pilot study.

Some imprinted genes have key functions influencing the proper embryonic growth and development by forming "imprinted gene network", including *GRB10* and *MEST* as the core molecules [13]. *GRB10*, as an important growth-limiting factor, has a wide range of effects on embryonic development. Maternal *GRB10* knockout results in embryonic overgrowth, and the change of ICR of *GRB10* gene results in obvious dysplasia [21, 22]. Previous studies have found an increase methylation of *GRB10* in fetal samples of spontaneous abortion [23]. It has been found that the *Ddc* gene in mice, involved in the development of trabecular cardiomyocytes of the embryonic and neonatal heart, displays tight conserved linkage with the methylated *GRB10* gene [24]. In the present study, we found that the gDMR methylation of GRB10 imprinted gene in CHD was increased except for valvar defects. It is hinted the methylation disorder of *GRB10* may be involved in the development of heart through the interaction with *Ddc*. Hypermethylation of *GRB10* might inhibit its expression and regulate the growth through insulin pathway such as *IGF2*, potentially leading to cardiac dysplasia. *MEST* is a maternal imprinted gene and widely expressed throughout the embryonic period [25]. *Mest*-knockout mice exhibited embryonic and placental growth retardation and postnatal growth inhibition, while loss of the imprinted gene resulted in postnatal weight gain and multiple organ hypertrophy [26, 27]. King et al. have found that *Mest* specifically expressed myocardial trabeculae in developing atria and ventricles of mice. *Mest*-knockout mice showed subtle changes of myocardial trabeculae, which displayed an increase in thickness and reduction in density of the compact myocardium, similar to that in human heart disease, which hinted *MEST* is closely related to heart disease [28]. The increased methylation of gDMR of *MEST* imprinted gene in CHD was also found in our data, especially in ASD and VSD. Potentially, *MEST* expression disorder via altered methylation of the gene might be involved in the occurrence of CHD by affecting the development of myocardial trabecula.

*PEG10*, a paternally expressed imprinted gene, is expressed in embryonic tissues and placenta, and participates in cell proliferation, differentiation and apoptosis [29, 30]. The *Peg10*-knockout mice exhibited early embryo death and placental defects [31]. A study has found that the expression of *PEG10* in the heart was very low, indicating that trace *PEG10* could maintain the normal development of the heart [29]. On the contrary, we found abnormal hypomethylation of *PEG10* in CHD. Overexpression of *PEG10* may be involved in the invasion and metastasis of malignant tumors, such as hepatocellular carcinoma and endometrial cancer etc., through the epithelial mesenchymal transition (EMT) [32, 33]. Therefore, the low methylation of *PEG10* might cause abnormal elevation of expression, and induce abnormal migration of related cells through EMT pathway, potentially leading to abnormal cardiac development.

*INPP5F* encodes inositol 1,4,5-trisphosphate (InsP3) 5-phosphatase, which is an important functional endogenous regulator. It has been found that *INPP5F* regulated the size of cardiac myocytes and cardiac stress response, increased the hypertrophy and activation of the fetal gene program in the stress response in *Inpp5f*-knockout mice, and decreased the hypertrophic response in *Inpp5f*-overexpressed mice [34, 35]. *INPP5F* can be used as a negative feedback regulator of insulin signal and downregulation of *INPP5F* in diabetes mellitus has cardioprotective effect [36]. Consistent with previous studies, we found that INPP5F methylation was reduced in a variety of CHDs and increased CHD risk, suggesting that INPP5F might be a protective factor for cardiac development.

*MEG3* is a maternal-expressed imprinted gene that is expressed in many normal tissues. It inhibits the proliferation of tumor cells in vitro. It also interacts with tumor suppressor p53 to regulate the expression of p53 target genes [37]. Consistent with this effect, *MEG3* has an impact on cardiac remodeling induced by pathological hypertrophy by regulating the binding of p53 to other gene promoters [38]. Other studies have shown that *MEG3* participates in the development of myocardial fibrosis and prevents myocardial remodeling by regulating the production of MMP-2 by CFs in vitro and in vivo [39]. In consistency to previous studies, our results also found that methylation of *MEG3* was reduced and associated with the increased risk of CHD.

We also detected hypomethylation of gDMRs of other 3 imprinted genes including *NAP1L5*, *PLAGL1* and *NESP* in CHD. Stratified analysis revealed that different CHDs showed specific methylation patterns, such as hypomethylation of the 3 genes in ASD and VSD, hypomethylation of *NAP1L5* and *NESP* in valvular malformation, hypomethylation of *NESP* in thoracic vascular malformation, and no significant change in ToF.

As a pilot study, it is subject to a couple of limitations. First, we studied a small and heterogeneous group of patients with CHD. A large sample size is warranted to identify the methylation characteristics and risk factors of imprinted genes in relation to the types of CHD. Second, the causal relationship between methylation alterations of imprinted genes and CHD remains to be explored in further experimental studies to examine their potential impact on RNA expression and CHD development on cellular and organ levels in animal models.

## Conclusion

Methylation of gDMRs of 8 imprinted genes was altered in white blood cells in patients with CHD. Further experiments are warranted to identify the methylation characteristics of imprinted genes in different types of CHD and clarify the pathogenesis mechanism of imprinted genes in CHD.

## Supplementary information

**Additional file 1: Fig. S1.** Analysis of methylation level of GRB10. **a** The mean methylation of gDMR of GRB10 between CHD group and control group. **b** Methylation level of specific CpG site in GRB10 between CHD group and control group. CpG sites included numbered 1–4 from the 5′ end to the 3′ end. *P < 0.05; **P < 0.01.

**Additional file 2: Fig. S2.** Analysis of methylation level of PEG10. **a** The mean methylation of gDMR of PEG10 between CHD group and control group. **b** Methylation level of specific CpG site in PEG10 between CHD group and control group. CpG sites included numbered 1–12 from the 5′ end to the 3′ end. *P < 0.05; **P < 0.01.

**Additional file 3: Fig. S3.** Analysis of methylation level of MEST. **a** The mean methylation of gDMR of MEST between CHD group and control group. **b** Methylation level of specific CpG site in MEST between CHD group and control group. CpG sites included numbered 1–26 from the 5′ end to the 3′ end. *P < 0.05; **P < 0.01.

**Additional file 4: Fig. S4.** Analysis of methylation level of NAP1L5. **a** The mean methylation of gDMR of NAP1L5 between CHD group and control group. **b** Methylation level of specific CpG site in NAP1L5 between CHD group and control group. CpG sites included numbered 1–7 from the 5′ end to the 3′ end. *P < 0.05; **P < 0.01.

**Additional file 5: Fig. S5.** Analysis of methylation level of INPP5F. **a** The mean methylation of gDMR of INPP5F between CHD group and control group. **b** Methylation level of specific CpG site in INPP5F between CHD group and control group. CpG sites included numbered 1–33 from the 5′ end to the 3′ end. *P < 0.05; **P < 0.01.

**Additional file 6: Fig. S6.** Analysis of methylation level of PLAGL1. **a** The mean methylation of gDMR of PLAGL1 between CHD group and control group. **b** Methylation level of specific CpG site in PLAGL1 between CHD group and control group. CpG sites included numbered 1–30 from the 5′ end to the 3′ end. *P < 0.05; **P < 0.01.

**Additional file 7: Fig. S7.** Analysis of methylation level of NESP. **a** The mean methylation of gDMR of NESP between CHD group and control group. **b** Methylation level of specific CpG site in NESP between CHD group and control group. CpG sites included numbered 1–22 from the 5′ end to the 3′ end. *P < 0.05; **P < 0.01.

**Additional file 8: Fig. S8.** Analysis of methylation level of MEG3. **a** The mean methylation of gDMR of MEG3 between CHD group and control group. **b** Methylation level of specific CpG site in MEG3 between CHD group and control group. CpG sites included numbered 1–7 from the 5′ end to the 3′ end. *P < 0.05; **P < 0.01.

**Additional file 9: Fig. S9.** Abnormal methylation level of the imprinted genes between multiple CHDs classification and control groups. **a** Abnormal methylation level of the imprinted genes between AVSD and control groups. **b** Abnormal methylation level of the imprinted genes between VHD and control groups. **c** Abnormal methylation level of the imprinted genes between ToF and control groups. **d** Abnormal methylation level of the imprinted genes between VM and control groups. AVSD, atrioventricular septal defect. *P < 0.05; **P < 0.01.

**Additional file 10: Table S1.** gDMR sequence information of 18 imprinted genes.

**Additional file 11: Table S2.** Primer sequence information of 18 imprinted genes.

**Additional file 12: Table S3.** The average methylation levels of 18 imprinted genes detected in CHD patients and healthy individuals.

**Additional file 13: Table S4.** CpG sites methylation level of 18 imprinted genes detected in CHD patients and healthy individuals.

**Additional file 14: Table S5.** CpG sites methylation level of 18 imprinted genes detected in CHD patients and healthy individuals.

**Additional file 15: Table S6.** CpG sites methylation level of 18 imprinted genes detected in CHD patients and healthy individuals.

**Additional file 16: Table S7.** CpG sites methylation level of 18 imprinted genes detected in CHD patients and healthy individuals.

**Additional file 17: Table S8.** CpG sites methylation level of 18 imprinted genes detected in CHD patients and healthy individuals.

**Additional file 18: Table S9.** CpG sites methylation level of 18 imprinted genes detected in CHD patients and healthy individuals.

**Additional file 19: Table S10.** CpG sites methylation level of 18 imprinted genes detected in CHD patients and healthy individuals.

**Additional file 20: Table S11.** CpG sites methylation level of 18 imprinted genes detected in CHD patients and healthy individuals.

**Additional file 21: Table S12.** CpG sites methylation level of 18 imprinted genes detected in CHD patients and healthy individuals.

**Additional file 22: Table S13.** CpG sites methylation level of 18 imprinted genes detected in CHD patients and healthy individuals.

**Additional file 23: Table S14.** CpG sites methylation level of 18 imprinted genes detected in CHD patients and healthy individuals.

**Additional file 24: Table S15.** CpG sites methylation level of 18 imprinted genes detected in CHD patients and healthy individuals.

**Additional file 25: Table S16.** CpG sites methylation level of 18 imprinted genes detected in CHD patients and healthy individuals.

**Additional file 26: Table S17.** CpG sites methylation level of 18 imprinted genes detected in CHD patients and healthy individuals.

**Additional file 27: Table S18.** CpG sites methylation level of 18 imprinted genes detected in CHD patients and healthy individuals.

**Additional file 28: Table S19.** CpG sites methylation level of 18 imprinted genes detected in CHD patients and healthy individuals.

**Additional file 29: Table S20.** CpG sites methylation level of 18 imprinted genes detected in CHD patients and healthy individuals.

**Additional file 30: Table S21.** CpG sites methylation level of 18 imprinted genes detected in CHD patients and healthy individuals.

## Data Availability

The dataset generated and analyzed during the present study are presented in the supplementary files.

## Abbreviations

ASD: Atrial septal defect
CHD: Congenital heart disease
gDMRs: Germline differential methylation regions
ToF: Tetralogy of Fallot
VSD: Ventricular septal defect
