# Supplementary figures and images for: DNA methylation abnormalities of imprinted genes in congenital heart disease: a pilot study
Source: BMC Med Genomics. 2021 Jan 6;14:4. doi: 10.1186/s12920-020-00848-0 (PMC7789576; doi:10.1186/s12920-020-00848-0)

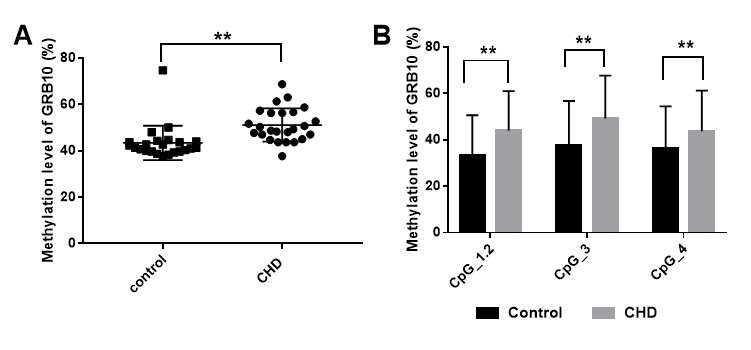

Supplement: Supplementary file 1 — Additional file 1: Fig. S1. Analysis of methylation level of GRB10. a The mean methylation of gDMR of GRB10 between CHD group and control group. b Methylation level of specific CpG site in GRB10 between CHD group and control group. CpG sites included numbered 1–4 from the 5′ end to the 3′ end. *P < 0.05; **P < 0.01. [file 12920_2020_848_MOESM1_ESM.tif]

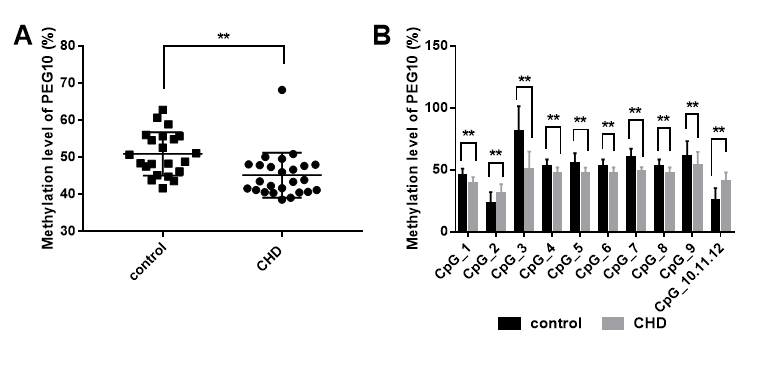

Supplement: Supplementary file 2 — Additional file 2: Fig. S2. Analysis of methylation level of PEG10. a The mean methylation of gDMR of PEG10 between CHD group and control group. b Methylation level of specific CpG site in PEG10 between CHD group and control group. CpG sites included numbered 1–12 from the 5′ end to the 3′ end. *P < 0.05; **P < 0.01. [file 12920_2020_848_MOESM2_ESM.tif]

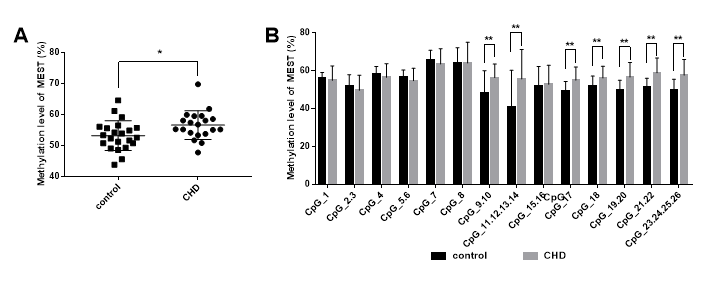

Supplement: Supplementary file 3 — Additional file 3: Fig. S3. Analysis of methylation level of MEST. a The mean methylation of gDMR of MEST between CHD group and control group. b Methylation level of specific CpG site in MEST between CHD group and control group. CpG sites included numbered 1–26 from the 5′ end to the 3′ end. *P < 0.05; **P < 0.01. [file 12920_2020_848_MOESM3_ESM.tif]

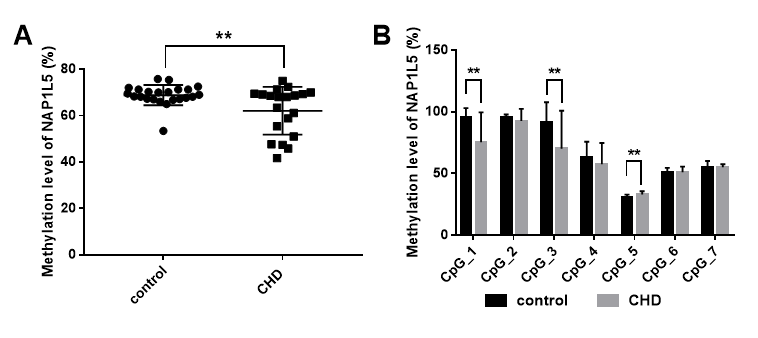

Supplement: Supplementary file 4 — Additional file 4: Fig. S4. Analysis of methylation level of NAP1L5. a The mean methylation of gDMR of NAP1L5 between CHD group and control group. b Methylation level of specific CpG site in NAP1L5 between CHD group and control group. CpG sites included numbered 1–7 from the 5′ end to the 3′ end. *P < 0.05; **P < 0.01. [file 12920_2020_848_MOESM4_ESM.tif]

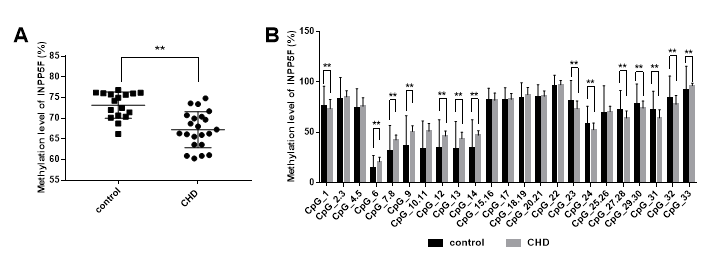

Supplement: Supplementary file 5 — Additional file 5: Fig. S5. Analysis of methylation level of INPP5F. a The mean methylation of gDMR of INPP5F between CHD group and control group. b Methylation level of specific CpG site in INPP5F between CHD group and control group. CpG sites included numbered 1–33 from the 5′ end to the 3′ end. *P < 0.05; **P < 0.01. [file 12920_2020_848_MOESM5_ESM.tif]

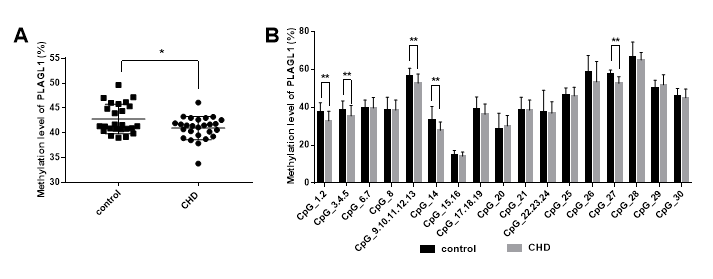

Supplement: Supplementary file 6 — Additional file 6: Fig. S6. Analysis of methylation level of PLAGL1. a The mean methylation of gDMR of PLAGL1 between CHD group and control group. b Methylation level of specific CpG site in PLAGL1 between CHD group and control group. CpG sites included numbered 1–30 from the 5′ end to the 3′ end. *P < 0.05; **P < 0.01. [file 12920_2020_848_MOESM6_ESM.tif]

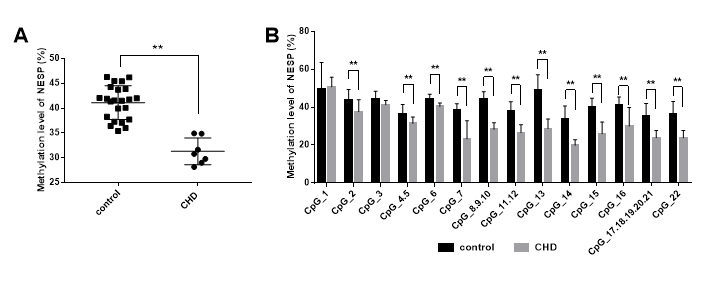

Supplement: Supplementary file 7 — Additional file 7: Fig. S7. Analysis of methylation level of NESP. a The mean methylation of gDMR of NESP between CHD group and control group. b Methylation level of specific CpG site in NESP between CHD group and control group. CpG sites included numbered 1–22 from the 5′ end to the 3′ end. *P < 0.05; **P < 0.01. [file 12920_2020_848_MOESM7_ESM.tif]

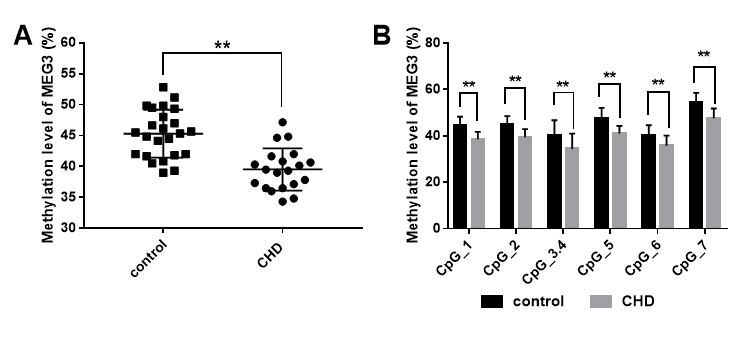

Supplement: Supplementary file 8 — Additional file 8: Fig. S8. Analysis of methylation level of MEG3. a The mean methylation of gDMR of MEG3 between CHD group and control group. b Methylation level of specific CpG site in MEG3 between CHD group and control group. CpG sites included numbered 1–7 from the 5′ end to the 3′ end. *P < 0.05; **P < 0.01. [file 12920_2020_848_MOESM8_ESM.tif]

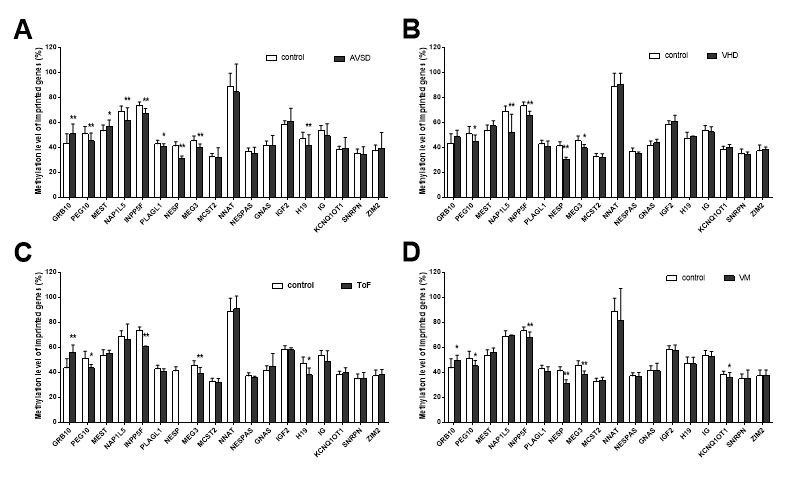

Supplement: Supplementary file 9 — Additional file 9: Fig. S9. Abnormal methylation level of the imprinted genes between multiple CHDs classification and control groups. a Abnormal methylation level of the imprinted genes between AVSD and control groups. b Abnormal methylation level of the imprinted genes between VHD and control groups. c Abnormal methylation level of the imprinted genes between ToF and control groups. d Abnormal methylation level of the imprinted genes between VM and control groups. AVSD, atrioventricular septal defect. *P < 0.05; **P < 0.01. [file 12920_2020_848_MOESM9_ESM.tif]
