# Supplementary material for: DNA methylation abnormalities of imprinted genes in congenital heart disease: a pilot study
Source: BMC Med Genomics. 2021 Jan 6;14:4. doi: 10.1186/s12920-020-00848-0 (PMC7789576; doi:10.1186/s12920-020-00848-0)
Supplement: Supplementary file 10 — Additional file 10: Table S1. gDMR sequence information of 18 imprinted genes. [file 12920_2020_848_MOESM10_ESM.docx]

**Table S1 DMR sequence information of 18 imprinted genes**

| **Source** | **Gene ID** | **DMR Position (UCSC hg18)** |
| --- | --- | --- |
| maternal imprinting | GRB10 | chr7: 50,817,247- 50,818,365 |
|  | PEG10 | chr7: 94,124,118- 94,124,493 |
|  | MEST | chr7: 129,919,302- 129,919,592 |
|  | NAP1L5 | chr4: 89,837,390- 89,839,620 |
|  | INPP5F | chr10: 121,567,520- 121,568,375 |
|  | PLAGL1 | chr6: 144,371,101- 144,371,640 |
|  |  | chr6: 144,370,610- 144,371,540 |
|  | MCTS2 | chr20: 29,598,738- 29,598,953 |
|  | NNAT | chr20: 35,580,532- 35,584,472 |
|  | NESPAS | chr20: 56,863,391- 56,864,195 |
|  | GNAS | chr20: 56,898,138- 56,898,355 |
|  | KCNQ1OT1 | chr11: 2,677,111-   2,678,664 |
|  |  | chr11: 2,677,602-   2,678,664 |
|  |  | chr11: 2,677,672-   2,678,044 |
|  |  | chr11: 2,677,757-   2,678,763 |
|  | SNRPN | chr15: 22,751,027- 22,751,436 |
|  |  | chr15: 22,751,106- 22,751,320 |
|  |  | chr15: 22,750,755- 22,750,990 |
|  | ZIM2 | chr19: 62,043,540- 62,043,985 |
| paternal imprinting | NESP | chr20: 56,848,246- 56,850,403 |
|  | GTL2/DLK1 | [chr14: 100,345,426- 100,345,735](http://genome.ucsc.edu/cgi-bin/hgTracks?db=hg18&position=chr14:100345426-100345735) |
|  | (MEG3, IG) | [chr14: 100,346,924- 100,347,309](http://genome.ucsc.edu/cgi-bin/hgTracks?db=hg18&position=chr14:100346924-100347309) |
|  |  | [chr14: 100,346,990- 100,347,246](http://genome.ucsc.edu/cgi-bin/hgTracks?db=hg18&position=chr14:100346990-100347246) |
|  | H19/IGF2 | chr11: 1,975,944- 1,976,304 |
|  |  | chr11: 1,977,410- 1,977,914 |
|  |  | chr11: 1,977,575- 1,978,014 |
|  |  | chr11: 1,977,644- 1,980,075 |
|  |  | chr11: 1,977,647- 1,977,977 |
|  |  | chr11: 1,977,647- 1,977,970 |
|  |  | chr11: 1,977,647- 1,977,878 |
