# Supplementary material for: DNA methylation abnormalities of imprinted genes in congenital heart disease: a pilot study
Source: BMC Med Genomics. 2021 Jan 6;14:4. doi: 10.1186/s12920-020-00848-0 (PMC7789576; doi:10.1186/s12920-020-00848-0)
Supplement: Supplementary file 11 — Additional file 11: Table S2. Primer sequence information of 18 imprinted genes. [file 12920_2020_848_MOESM11_ESM.docx]

**Table S2 Primer sequence information of 18 imprinted genes**

| **Gene ID** | **Primer name** | **Primer sequence** |
| --- | --- | --- |
| GRB10 | GRB10_L | aggaagagagAGGAATTTTAGGATTAAATTTATGTGA |
|  | GRB10_R | cagtaatacgactcactatagggagaaggctTTCTTCAAAATTACCATAAAAACCA |
| PEG10 | PEG10-L | aggaagagagGGGAAATTAAGTTTTAAAGGTTTGG |
|  | PEG10-R | cagtaatacgactcactatagggagaaggctCCAATTATAACTCCCATCAATTT |
| MEST | MEST_1L | aggaagagagTAGTTAAAGGAATTAGGAAGGGAAAT |
|  | MEST_1R | cagtaatacgactcactatagggagaaggctCTAAAACCAAAACTACTTCCCACAC |
|  | MEST_2L | cagtaatacgactcactatagggagaaggctAATACCTAAATCTTAAAATCCTAAACTACACC |
|  | MEST_2R | cagtaatacgactcactatagggagaaggctAATACCTAAATCTTAAAATCCTAAACTACACC |
| NAP1L5 | NAP1L5_1L | aggaagagagTGTTTTTAGGGGAGTTTTGTTGA |
|  | NAP1L5_1R | cagtaatacgactcactatagggagaaggctCCAAACCCTTCTACAAATACCAA |
|  | NAP1L5_2L | aggaagagagTTGGTATTTGTAGAAGGGTTTGG |
|  | NAP1L5_2R | cagtaatacgactcactatagggagaaggctCACTTAACTAACTTCCCACTATCAA |
| INPP5F | INPP5F_1L | aggaagagagGTTTTGAGATTGGAGAGGAAATTA |
|  | INPP5F_1R | cagtaatacgactcactatagggagaaggctAACCACTCCATTACCACTACTAAACC |
|  | INPP5F_2L | aggaagagagGTTGGGTGTAGGTAGTTGTTTGAGT |
|  | INPP5F_2R | cagtaatacgactcactatagggagaaggctCTTTAACCATAAACCCTACAAAAAAA |
|  | INPP5F_3L | aggaagagagTGTATGGGAGAAGGGTTAGGATAG |
|  | INPP5F_3R | cagtaatacgactcactatagggagaaggctAATCCCAACAAAAAAATAAACCTAACT |
|  | INPP5F_4L | aggaagagagGAAGTAAGTTGGGAATATTATTTGG |
|  | INPP5F_4R | cagtaatacgactcactatagggagaaggctCTATCCTAACCCTTCTCCCATACA |
| PLAGL1 | PLAGL1_1L | aggaagagagGGGTTGAATGATAAATGGTAGATG |
|  | PLAGL1_1R | cagtaatacgactcactatagggagaaggctACCTTAACTTTACCCCCACC |
|  | PLAGL1_2L | cagtaatacgactcactatagggagaaggctAAAAACAATAAAAATTCCCTTCAAT |
|  | PLAGL1_2R | aggaagagagTATTTTTGTGGGGATGGAGGAATTA |
| NESP | NESP_1L | 5'-aggaagagagGTTAAATTGGGGAGTTTGAGGG-3' |
|  | NESP_1R | 5'-cagtaatacgactcactatagggagaaggctCAACAAACCTTTAACCACCAAAAC-3' |
|  | NESP_2L | 5'-aggaagagagTTTTAAATAAGTTGGTTTTTTTAGGTGT-3' |
|  | NESP_2R | 5'-cagtaatacgactcactatagggagaaggctCTTAATTATCAACTCTAAAACCTAAACTCC-3' |
| MEG3 | MEG3_L | aggaagagagTTGTGTTTGAATTTATTTTGTTT |
|  | MEG3_R | cagtaatacgactcactatagggagaaggctCCCCAAATTCTATAACAAATTACT |
| MCST2 | MCST2_1L | aggaagagagGGTTTTTTTGTAGGGGATTTTAT |
|  | MCST2_1R | cagtaatacgactcactatagggagaaggctACAATTAAACACACTTTCCTTTTCA |
|  | MCST2_2L | aggaagagagGGTGGAGTTTTTTGTAGGAAGGT |
|  | MCST2_2R | cagtaatacgactcactatagggagaaggctTTTCTAACTTATCAAACCCTACTATTTCAA |
| NNAT | NNAT_L | aggaagagagTTTTTTTTGTAGGAAGAATTTTTTG |
|  | NNAT_R | cagtaatacgactcactatagggagaaggctAAACCCACAACTTAAATATACCTCA |
| NESPAS | NESPAS_1L | 5'-aggaagagagTTTTTAAGGTTAAGAAGGTATTTTTGG-3' |
|  | NESPAS_1R | 5'-cagtaatacgactcactatagggagaaggctCTTTAACTAAACCCCCTCCTCC-3' |
| NESPAS | NESPAS_2L | 5'-aggaagagagTTGTTAGAAAGTTTTAGGGAGGGAT-3' |
|  | NESPAS_2R | 5'-cagtaatacgactcactatagggagaaggctCCACCAACCTAACCAAAAAAATC-3' |
| GNAS | GNAS_L | aggaagagagTGTTTTTTGGTTTGTTTTTGTTTTT |
|  | GNAS_R | cagtaatacgactcactatagggagaaggctCATAACCATCTTCAACATAATAACCC |
| IGF2 | IGF2_1L | aggaagagagTTTTAGGGTTTGGTTTGAGGGTA |
|  | IGF2_1R | cagtaatacgactcactatagggagaaggctTCCAAAAAAACCAAATCACAACTAC |
|  | IGF2_2L | aggaagagagGGTTTTTGTTTTTGGTTAAGAGGT |
|  | IGF2_2R | cagtaatacgactcactatagggagaaggctCAACTATAACCTAACCCTCCTAAAA |
|  | IGF2_3L | aggaagagagATTGTTGGTTATTTTTGGGGG |
|  | IGF2_3R | cagtaatacgactcactatagggagaaggctTAAACAAATTCTTCCAATATAACACCT |
|  | IGF2_4L | aggaagagagTTATTTAGGGTGGTGTTTGTGG |
|  | IGF2_4R | cagtaatacgactcactatagggagaaggctACCTAACACTAAAAATAAAAAATACACAC |
| H19 | H19_1L | aggaagagagTATGGGTATTTTTGGAGGTTTTTTT |
|  | H19_1R | cagtaatacgactcactatagggagaaggctAACTTAAATCCCAAACCATAACACT |
|  | H19_2L | aggaagagagTTTTTATTAAAGGTTAAGGTGGTGAT |
|  | H19_2R | cagtaatacgactcactatagggagaaggctCAAAACAAAATCCCCACAACC |
| IG | IG_L | aggaagagagGTTAAGAGTTTGTGGATTTGTGAGAAATG |
|  | IG_R | cagtaatacgactcactatagggagaaggctACAATTCCTACTACAAAATTTCAACA |
| KCNQ1OT1 | KCNQ1OT1_L | aggaagagagGGTTTTTGTGATTTAGGTTTTTGTTT |
|  | KCNQ1OT1_R | cagtaatacgactcactatagggagaaggctAACATACCAAACCACCCACC |
| SNRPN | SNRPN_L | aggaagagagTGTGGGGTTTTAGGGGTTTAG |
|  | SNRPN_R | cagtaatacgactcactatagggagaaggctCTCCCCAAACTATCTCTTAAAAAAA |
| ZIM2 | ZIM2_L | aggaagagagGGTGAGGTTGTTGATTGGTTAGTAT |
|  | ZIM2_R | cagtaatacgactcactatagggagaaggctCCACCAACCCAAAATAAACAT |
