# Supplementary material for: DNA methylation abnormalities of imprinted genes in congenital heart disease: a pilot study
Source: BMC Med Genomics. 2021 Jan 6;14:4. doi: 10.1186/s12920-020-00848-0 (PMC7789576; doi:10.1186/s12920-020-00848-0)
Supplement: Supplementary file 12 — Additional file 12: Table S3. The average methylation levels of 18 imprinted genes detected in CHD patients and healthy individuals. [file 12920_2020_848_MOESM12_ESM.pdf]

Table S3.1 The average methylation levels of 18 imprinted genes detected in CHD patients and healthy individuals

Groups	SampleID	GRB10	PEG10	MEST	NAP1L5	INPP5F	PLAGL1
	1	0.4133	0.4520		0.6900		0.4082
	2	0.3933	0.4880	0.4557	0.6814		
	3	0.4433	0.4620	0.5079	0.7029	0.7423	0.4535
	4	0.4367		0.5393	0.6700	0.7604	0.4965
	5	0.4133	0.4380	0.5121	0.7200	0.7252	0.4082
	6	0.4267	0.5260	0.4936	0.6729	0.7143	0.4441
	7				0.7129	0.7565	0.3988
	8	0.4267	0.5460	0.5164	0.6814	0.6874	
	9		0.4170	0.4857		0.7068	0.4088
	10	0.4400	0.4830	0.4907	0.7129		0.4171
	11	0.4367	0.4820			0.7014	0.4159
	12	0.3967		0.5607	0.6771	0.6622	0.4394
	13	0.4067	0.4360	0.5486	0.7000		0.4129
Control	14	0.3967	0.5110	0.5650	0.6686	0.7495	0.4706
	15	0.4000	0.6280	0.4386	0.7129	0.7039	0.4094
	16	0.4100		0.5229		0.7200	0.4582
	17	0.3833	0.5490	0.5257	0.7257		0.4041
	18	0.4800	0.4480	0.5914	0.6600		0.4135
	19	0.3867		0.6114		0.7574	0.3900
	20						
	21	0.3767	0.4840	0.6457	0.6500		0.4606
	22	0.4233	0.5070	0.5421	0.6714	0.7617	0.4135
	23	0.4033	0.5570	0.5571	0.6957	0.7600	0.4618
	24		0.5570	0.5557	0.5343	0.7622	0.4488
	25	0.5000	0.5600	0.5079	0.7543		0.4076
	26	0.4467	0.4750	0.5336	0.6829		0.3941
	27	0.7467	0.6070		0.7586		0.4718
	28		0.5890		0.7014	0.7683	0.3918
	1	0.6133	0.4810	0.5950	0.7129		0.4059
	2	0.4500	0.3860	0.5957	0.6343		0.4153
	3	0.5733	0.3910		0.6929		0.4171
	4	0.4367	0.4040	0.5529	0.4586	0.6613	0.4024
	5	0.4367	0.4340	0.5807	0.5543	0.6557	0.3953
	6	0.4800	0.4670	0.5993		0.6361	0.4182
	7	0.4867	0.4960	0.5807	0.6943	0.6630	0.4194
	8	0.4933	0.4770	0.5379	0.6857	0.7361	0.3876
	9		0.4800	0.4779	0.5886	0.7483	
	10	0.5867		0.5521	0.4743	0.6804	0.4153
	11	0.5167		0.5336		0.7343	0.3788
	12	0.4700	0.4060	0.5857		0.6730	0.4112
	13	0.4467	0.4070	0.5507	0.6114	0.6030	0.4324
CHD	14	0.6867	0.4740			0.6635	0.3929
	15	0.5067	0.5090				0.4265
	16	0.5633	0.5010		0.4171	0.6109	0.4071
	17	0.4833	0.4350		0.4771	0.6596	0.4035
	18	0.4367	0.4120	0.6186	0.6871	0.6091	0.3382
	19		0.4120	0.5679	0.6800		0.4300
	20	0.5667	0.4790	0.5521	0.7000	0.6965	0.4259
	21	0.4700	0.4160	0.6979		0.6361	0.4300

22	0.5633	0.6820		0.5100		0.4171
23	0.5033	0.4600	0.5171	0.7243	0.7074	0.4329
24	0.4767	0.4230	0.5414		0.7039	0.4612
25	0.3767	0.4170	0.5086	0.6800	0.6870	0.4141
26	0.6300	0.4390	0.5743	0.6914	0.6091	0.3894
27	0.5267	0.4050		0.7500	0.6996	0.3853

Table S3.2 The average methylation levels of 18 imprinted genes detected in CHD patients and healthy individuals

Groups	SampleID	NESP	MEG3	MCST2	NNAT	NESPAS	GNAS
Control	1	0.3829		0.3171	0.7900		
	2	0.4136	0.5283	0.3257	0.9667	0.3150	
	3	0.4157	0.4167	0.2857		0.3875	0.4079
	4	0.3643	0.4983	0.3400	0.9367	0.3842	0.4593
	5		0.4450	0.2757	0.9467	0.3125	
	6		0.4667	0.3214	0.9300	0.3233	0.4200
	7		0.4950	0.3114	0.7933		0.3962
	8	0.3729	0.5117	0.3029	0.6967	0.3833	0.4371
	9	0.3771	0.4200				0.3700
	10	0.4150	0.4200	0.3400	0.9433	0.3675	0.4350
	11			0.3214			
	12	0.4386	0.3900	0.3271	0.9200	0.3633	0.4400
	13	0.3543	0.4083	0.2829	0.7433		0.3679
	14	0.4207	0.4933	0.3157	0.9367		0.3800
	15	0.4007	0.4700	0.2829		0.3675	0.4200
	16	0.3707	0.4617	0.3743			0.4536
	17	0.4200	0.3933		0.9533	0.3775	0.3964
	18	0.4429	0.4183	0.3386			0.3743
	19	0.4550	0.4533	0.3186		0.3658	0.4893
	20						
	21	0.4621	0.4983	0.3414	0.9267	0.3925	0.3964
	22	0.3600	0.4800	0.3486	0.9533	0.3742	0.3964
	23	0.4543	0.4050	0.3114	0.9567	0.3775	0.4664
	24	0.4193	0.4417	0.3500	0.5833	0.3867	0.4700
	25	0.4629	0.4567	0.2929	0.8700	0.3867	0.3786
	26	0.3993	0.4483	0.3371	0.9300	0.3575	0.4236
	27	0.4371	0.4550	0.3686	0.9800	0.4083	0.4193
	28	0.4179		0.3757	0.9633	0.3983	0.3979
CHD	1			0.3243	0.8860		0.4172
	2		0.4717	0.0280	0.1076	0.3583	0.0346
	3		0.4033	0.3392	0.9194	0.3458	0.6768
	4		0.4067	0.0337	0.1383	0.3400	0.0609
	5		0.3650	0.3327	0.8899	0.3533	0.4111
	6	0.3079	0.3783	0.3443	0.9133	0.3842	0.3679
	7		0.4200	0.3343	0.9433		
	8	0.3492		0.3243	0.9333	0.3775	0.4400
	9		0.3483	0.4443		0.3492	0.3800
	10		0.4483	0.3329	0.7500		0.3693
	11		0.3933	0.3443	0.3167	0.3617	0.2871
	12		0.3717	0.3629	0.9567		0.4250
	13	0.2979	0.3650	0.3671	0.9533	0.3567	0.3721
	14		0.4017	0.3229	0.9167		0.4021
	15		0.3600	0.3600	0.9700	0.4150	0.4736
	16			0.2929	0.9267		0.4036
	17	0.3486		0.3057	0.8933	0.3408	0.4043
	18	0.2900	0.4167	0.3271	0.9467	0.3658	0.4329
	19		0.4467		0.7600	0.3400	
	20	0.2815		0.3414		0.3183	0.4000
	21			0.3429	0.9433		0.5207

22	0.3164	0.3900	0.3429	0.9367		0.3821
23		0.3733	0.2843	0.9600		0.4486
24			0.2900	0.7667		0.4364
25		0.3950	0.3200	0.8667		0.3700
26		0.4083	0.3429	0.9533		0.3877
27		0.3433		0.9600		0.5914

Table S3.3 The average methylation levels of 18 imprinted genes detected in CHD patients and healthy individuals

Groups	SampleID	IGF2	H19	IG	KCNQ1OT1	SNRPN	ZIM2
	1	0.5925			0.3900	0.3050	0.3650
	2	0.5425	0.3275	0.5700	0.3800	0.3692	0.3175
	3		0.4475		0.3540		0.3675
	4			0.5870	0.3920	0.3275	0.3100
	5	0.6165	0.4958	0.5430	0.3840		0.2925
	6		0.5725	0.5070	0.3380	0.3464	0.3575
	7	0.5740	0.4858	0.5445	0.3960	0.2909	0.3525
	8	0.5665			0.4080	0.4175	0.4000
	9	0.5985	0.4575		0.3600	0.3350	
	10		0.4925	0.5700	0.3860	0.3542	0.3750
	11		0.4858		0.4020	0.3991	0.4325
	12	0.5490	0.4736	0.5495	0.3780	0.3108	0.3900
	13		0.5083	0.5400	0.3780	0.3750	0.3675
Control	14	0.5870				0.2818	0.3850
	15		0.4717		0.4375	0.3925	0.3825
	16	0.6040			0.3680	0.3255	0.5200
	17	0.6210	0.4442	0.5370		0.3392	0.3775
	18	0.6025	0.4408	0.5675	0.4200	0.3733	0.3350
	19			0.5325	0.3480	0.3025	0.3825
	20		0.4483				
	21	0.6180		0.5450	0.3660	0.3058	0.3600
	22		0.4050	0.6160	0.3660	0.3658	0.3550
	23	0.5645		0.4815	0.3960	0.3317	0.3150
	24	0.5825	0.4508	0.5025	0.3700	0.3658	0.3875
	25	0.5325	0.5650	0.4995	0.4380	0.4217	0.4450
	26	0.5340	0.4492	0.4920		0.3592	0.3375
	27	0.6170	0.5033		0.3900		0.3900
	28		0.4760	0.4720	0.3560	0.3683	
	1	0.5825	0.4701	0.5365	0.3834	0.3485	0.3720
	2	0.0302	0.0522	0.0384	0.0258	0.0385	0.0470
	3	0.9932	0.1667	0.1492	0.7139	0.4575	0.8633
	4	0.0416	0.0552	0.0413	0.0387	0.0546	0.0507
	5	0.5826	0.4437	0.5169	0.3799	0.3383	0.3696
	6	0.6680	0.4983	0.4990	0.3980	0.2883	0.3750
	7	0.5875	0.4042	0.4825	0.3900	0.4442	0.3050
	8		0.5258	0.6055	0.3360	0.3842	0.4075
	9	0.5650	0.4333	0.5370	0.3420	0.3475	0.2525
	10	0.5915	0.4183	0.5480	0.4100	0.3300	0.3450
	11	0.5425	0.4508	0.4920	0.2820	0.2633	0.3025
	12	0.5400		0.5145	0.4040	0.4792	
	13	0.5775	0.3725	0.4835	0.3820	0.3492	0.3250
CHD	14	0.6755		0.5645		0.3300	0.3425
	15			0.4800	0.3700	0.3158	0.3825
	16		0.4836	0.5655	0.4360	0.3692	0.3950
	17	0.5910	0.4583	0.5195	0.3380	0.2608	0.3500
	18	0.5765	0.4900		0.3940	0.3425	0.4000
	19	0.5840			0.4100		0.3575
	20	0.5845	0.4008	0.5140	0.3300	0.3242	0.4100
	21	0.5645			0.3480	0.3267	0.3925

22	0.5420	0.4208	0.5280	0.3900	0.2750	0.4775
23	0.5680			0.3440	0.3358	0.3700
24	0.6435		0.5265	0.3820	0.3408	0.3550
25			0.5390	0.3800	0.3542	0.4075
26		0.3391	0.4250	0.4275	0.3033	
27					0.4208	0.4275
