# Supplementary material for: DNA methylation abnormalities of imprinted genes in congenital heart disease: a pilot study
Source: BMC Med Genomics. 2021 Jan 6;14:4. doi: 10.1186/s12920-020-00848-0 (PMC7789576; doi:10.1186/s12920-020-00848-0)
Supplement: Supplementary file 13 — Additional file 13: Table S4. CpG sites methylation level of 18 imprinted genes detected in CHD patients and healthy individuals. [file 12920_2020_848_MOESM13_ESM.pdf]

Table S4 CpG sites methylation level of GRB10 in CHD patients and healthy individuals

Groups	SampleID	CpG_1.2	CpG_3	CpG_4
Control	1	0.38	0.46	0.4
	2	0.35	0.44	0.39
	3	0.4	0.48	0.45
	4	0.4	0.46	0.45
	5	0.39	0.44	0.41
	6	0.39	0.46	0.43
	7			
	8	0.4	0.45	0.43
	9			
	10	0.41	0.47	0.44
	11	0.43	0.42	0.46
	12	0.37	0.42	0.4
	13	0.37	0.44	0.41
	14	0.36	0.41	0.42
	15	0.34	0.44	0.42
	16	0.36	0.43	0.44
	17	0.37	0.43	0.35
	18	0.45	0.5	0.49
	19	0.35	0.44	0.37
	20			
	21	0.39	0.39	0.35
	22	0.38	0.43	0.46
	23	0.37	0.42	0.42
	24			
	25	0.49	0.49	0.52
	26	0.4	0.45	0.49
	27	0.75	0.78	0.71
	28			
CHD	1	0.58	0.66	0.6
	2	0.43	0.52	0.4
	3	0.49	0.65	0.58
	4	0.43	0.48	0.4
	5	0.44	0.49	0.38
	6	0.47	0.54	0.43
	7	0.49	0.49	0.48
	8	0.5	0.51	0.47
	9			
	10	0.6	0.59	0.57
	11	0.5	0.56	0.49
	12	0.43	0.51	0.47
	13	0.42	0.49	0.43
	14	0.69	0.7	0.67
	15	0.44	0.54	0.54
	16	0.54	0.59	0.56
	17	0.45	0.52	0.48

18	0.41	0.48	0.42
19			
20	0.53	0.56	0.61
21	0.46	0.55	0.4
22	0.5	0.64	0.55
23	0.44	0.55	0.52
24	0.48	0.55	0.4
25	0.36	0.43	0.34
26	0.63	0.64	0.62
27	0.59	0.55	0.44
