# Supplementary material for: DNA methylation abnormalities of imprinted genes in congenital heart disease: a pilot study
Source: BMC Med Genomics. 2021 Jan 6;14:4. doi: 10.1186/s12920-020-00848-0 (PMC7789576; doi:10.1186/s12920-020-00848-0)
Supplement: Supplementary file 14 — Additional file 14: Table S5. CpG sites methylation level of 18 imprinted genes detected in CHD patients and healthy individuals. [file 12920_2020_848_MOESM14_ESM.pdf]

Table S5.1 CpG sites methylation level of INPP5F in CHD patients and healthy individuals

Groups	SampleID	CpG_1	CpG_2.3	CpG_4.5	CpG_6	CpG_7.8	CpG_9	CpG_10.1[illegible]
Control	1							
	2							
	3	0.81	0.89	0.78	0.25	0.51	0.62	0.55
	4	0.83	0.91	0.79	0.28	0.52	0.6	0.61
	5	0.76	0.83	0.71	0.21	0.52	0.58	0.55
	6	0.8	0.91	0.81	0.24	0.45	0.57	0.55
	7	0.84	0.94	0.86	0.25	0.51	0.57	0.54
	8	0.67	0.76	0.69	0.26	0.42	0.55	0.61
	9	0.75	0.9	0.79	0.19	0.53	0.63	0.51
	10							
	11	0.73	0.85	0.69	0.24	0.51	0.59	0.53
	12	0.84	0.9	0.82	0.27	0.51	0.61	0.59
	13							
	14	0.81	0.88	0.78	0.18	0.49	0.61	0.52
	15	0.81	0.86	0.75	0.32	0.54	0.61	0.6
	16	0.82	0.9	0.77	0.21	0.52	0.58	0.52
	17							
	18							
	19	0.89	0.88	0.8	0.16	0.58	0.7	0.43
	20							
	21							
	22	0.83	0.89	0.82	0.26	0.5	0.59	0.61
	23	0.85	0.89	0.84	0.28	0.55	0.61	0.61
	24	0.85	0.92	0.84	0.26	0.54	0.6	0.58
	25							
	26							
	27							
	28	0.82	0.89	0.81	0.26	0.54	0.62	0.51
CHD	1							
	2							
	3	0.8	0.87	0.85	0.2	0.45	0.55	0.5
	4	0.71	0.85	0.74	0.22	0.4	0.45	0.67
	5	0.73	0.9	0.78	0.11	0.35	0.44	0.4
	6	0.74	0.87	0.81	0.17	0.38	0.44	0.51
	7	0.75	0.87	0.79	0.18	0.42	0.48	0.54
	8	0.76	0.94	0.85	0.3	0.51	0.62	0.64
	9	0.94	0.88	0.83	0.26	0.46	0.6	0.58
	10	0.69	0.84	0.73	0.21	0.44	0.52	0.5
	11	0.77	0.87	0.86	0.24	0.52	0.62	0.54
	12	0.7	0.8	0.7	0.25	0.42	0.49	0.57
	13	0.57	0.71	0.57	0.17	0.38	0.47	0.38
	14	0.68	0.84	0.74	0.18	0.35	0.44	0.48
	15							
	16	0.6	0.79	0.69	0.26	0.4	0.48	0.59

17	0.72	0.86	0.71	0.2	0.35	0.49	0.45
18	0.69	0.82	0.74	0.2	0.4	0.48	0.51
19							
20	0.88	0.96	0.95	0.13	0.46	0.56	0.38
21	0.77	0.92	0.78	0.24	0.47	0.48	0.55
22							
23	0.89	0.86	0.71	0.24	0.45	0.5	0.54
24	0.8	0.92	0.82	0.24	0.45	0.51	0.54
25	0.63	0.79	0.64	0.21	0.47	0.56	0.5
26	0.64	0.74	0.7	0.23	0.43	0.46	0.48
27	0.71	0.84	0.71	0.17	0.41	0.55	0.42

Table S5.2 CpG sites methylation level of INPP5F in CHD patients and healthy individuals

Groups	SampleID	CpG_12	CpG_13	CpG_14	CpG_15.1(	CpG_17	CpG_18.1(	CpG_20.2:
Control	1							
	2							
	3	0.56	0.54	0.57	0.86	0.91	0.93	0.93
	4	0.57	0.57	0.58	0.88	0.85	0.96	0.94
	5	0.54	0.53	0.57	1	0.87	0.71	0.99
	6	0.52	0.51	0.54	0.82	0.83	0.88	0.85
	7	0.56	0.52	0.55	0.89	0.87	0.91	0.91
	8	0.55	0.56	0.5	0.74	0.76	0.87	0.9
	9	0.56	0.56	0.59	0.81	0.76	0.76	0.74
	10							
	11	0.55	0.52	0.55	0.88	0.86	0.91	0.89
	12	0.59	0.56	0.56	0.51	0.46	0.38	0.47
	13							
	14	0.59	0.59	0.58	0.83	0.88	0.94	0.84
	15	0.53	0.54	0.57	0.6	0.64	0.69	0.7
	16	0.53	0.53	0.55	0.8	0.81	0.77	0.8
	17							
	18							
	19	0.61	0.61	0.64	0.71	0.75	0.97	0.84
	20							
	21							
	22	0.57	0.55	0.53	0.85	0.92	0.92	0.9
	23	0.59	0.61	0.57	0.89	0.9	0.93	0.91
	24	0.59	0.56	0.59	0.91	0.95	0.9	0.91
	25							
	26							
	27							
	28	0.59	0.49	0.58	0.92	0.93	0.92	0.93
CHD	1							
	2							
	3	0.5	0.46	0.52	0.89	0.88	0.93	0.92
	4	0.42	0.43	0.37	0.84	0.85	0.87	0.89
	5	0.37	0.3	0.48	0.87	0.87	0.93	0.89
	6	0.46	0.45	0.45	0.77	0.79	0.87	0.82
	7	0.43	0.42	0.48	0.8	0.81	0.89	0.88
	8	0.55	0.58	0.56	0.88	0.87	0.97	0.9
	9	0.54	0.53	0.5	0.89	0.83	0.88	0.88
	10	0.46	0.46	0.47	0.88	0.88	0.94	0.89
	11	0.56	0.51	0.58	0.83	0.87	0.88	0.9
	12	0.49	0.44	0.45	0.83	0.81	0.93	0.86
	13	0.42	0.42	0.41	0.78	0.77	0.82	0.83
	14	0.39	0.45	0.45	0.84	0.85	0.89	0.87
	15							
	16	0.46	0.44	0.44	0.74	0.72	0.81	0.79

17	0.41	0.35	0.43	0.75	0.81	0.79	0.84
18	0.45	0.43	0.47	0.69	0.7	0.76	0.76
19							
20	0.5	0.4	0.49	0.85	0.88	0.86	0.85
21	0.43	0.46	0.48	0.65	0.73	0.71	0.74
22							
23	0.49	0.45	0.51	0.78	0.8	0.92	0.9
24	0.52	0.5	0.45	0.85	0.87	0.89	0.88
25	0.45	0.47	0.48	0.93	0.9	0.98	0.9
26	0.44	0.33	0.45	0.76	0.8	0.79	0.76
27	0.45	0.39	0.47	0.9	0.91	0.93	0.92

Table S5.3 CpG sites methylation level of INPP5F in CHD patients and healthy individuals

Groups	SampleID	CpG_22	CpG_23	CpG_24	CpG_25.26	CpG_27.28	CpG_29.30	CpG_31
	1							
	2							
	3	1	0.91	NA	0.5	0.77	0.79	0.77
	4	1	0.86	0.68	0.76	0.8	0.83	0.78
	5	1	0.84	0.54	0.76	0.67	0.82	0.73
	6	1	0.8	0.51	0.8	0.7	0.75	0.75
	7	1	0.87	0.72	0.76	0.77	0.87	0.79
	8	1	0.77	0.75	0.65	0.67	0.7	0.65
	9	0.92	0.79	NA	0.78	0.7	0.76	0.7
	10							
	11	1	0.86	NA	0.07	0.77	0.8	0.78
	12	0.58	0.87	0.7	0.78	0.8	0.81	0.8
	13							
Control	14	1	0.87	NA	NA	0.83	0.85	0.78
	15	0.8	0.88	0.57	0.86	0.76	0.91	0.72
	16	1	0.85	0.54	0.84	0.74	0.86	0.75
	17							
	18							
	19	1	0.91	0.64	0.85	0.84	0.87	0.84
	20							
	21							
	22	1	0.89	0.64	0.87	0.82	0.87	0.81
	23	1	0.86	0.58	0.8	0.78	0.83	0.75
	24	1	0.84	0.61	0.82	0.78	0.81	0.78
	25							
	26							
	27							
	28	0.97	0.88	0.62	0.92	0.86	0.89	0.82
	1							
	2							
	3	1	0.79	0.57	0.75	0.71	0.79	0.73
	4	1	0.69	0.46	0.72	0.6	0.72	0.61
	5	1	0.71	0.53	0.74	0.62	0.72	0.62
	6	0.92	0.66	0.46	0.67	0.58	0.61	0.56
	7	1	0.72	0.5	0.67	0.58	0.68	0.63
	8	1	0.8	0.54	0.73	0.67	0.79	0.68
	9	1	0.88	0.63	0.81	0.78	0.87	0.8
	10	0.95	0.74	0.52	0.74	0.63	0.69	0.68
	11	1	0.82	0.67	0.77	0.73	0.81	0.72
	12	1	0.76	0.48	0.69	0.65	0.77	0.6
	13	0.97	0.64	0.46	0.65	0.55	0.66	0.58
CHD	14	1	0.72	0.52	0.7	0.63	0.77	0.63
	15							
	16	0.92	0.6	0.44	0.6	0.53	0.68	0.51

17	0.93	0.83	0.48	0.7	0.68	0.81	0.69
18	0.91	0.62	0.44	0.66	0.54	0.63	0.55
19							
20	1	0.77	0.51	0.71	0.66	0.79	0.69
21	0.85	0.64	0.59	0.67	0.58	0.75	0.62
22							
23	1	0.79	0.62	0.78	0.73	0.79	0.74
24	0.98	0.78	0.57	0.69	0.67	0.8	0.68
25	1	0.74	0.57	0.69	0.69	0.75	0.61
26	0.96	0.65	0.46	0.63	0.56	0.62	0.47
27	1	0.82	0.58	0.78	0.75	0.79	0.74

Table S5.4 CpG sites methylation level of INPP5F in CHD patients and healthy individuals

Groups	SampleID	CpG_32	CpG_33
	1		
	2		
	3	0.89	0.99
	4	0.89	1
	5	0.98	0.97
	6	0.86	0.98
	7	0.92	0.98
	8	0.81	0.97
	9	0.86	0.96
	10		
	11	0.85	1
	12	0.84	0.98
	13		
Control	14	0.92	0.97
	15	0.94	0.99
	16	0.88	0.99
	17		
	18		
	19	0.9	1
	20		
	21		
	22	0.92	0.96
	23	0.9	0.95
	24	0.9	0.99
	25		
	26		
	27		
	28	0.93	0.97
	1		
	2		
	3	0.84	0.99
	4	0.75	0.95
	5	0.77	0.95
	6	0.68	0.96
	7	0.78	0.95
	8	0.82	0.97
	9	0.87	0.97
	10	0.82	0.97
	11	0.85	0.97
	12	0.83	0.96
	13	0.7	0.96
CHD	14	0.85	0.99
	15		
	16	0.62	0.94

17	0.89	1
18	0.64	0.92
19		
20	0.78	0.96
21	0.62	0.9
22		
23	0.81	0.97
24	0.8	0.98
25	0.85	0.99
26	0.65	1
27	0.89	0.96
