# Supplementary material for: DNA methylation abnormalities of imprinted genes in congenital heart disease: a pilot study
Source: BMC Med Genomics. 2021 Jan 6;14:4. doi: 10.1186/s12920-020-00848-0 (PMC7789576; doi:10.1186/s12920-020-00848-0)
Supplement: Supplementary file 15 — Additional file 15: Table S6. CpG sites methylation level of 18 imprinted genes detected in CHD patients and healthy individuals. [file 12920_2020_848_MOESM15_ESM.pdf]

Table S6.1 CpG sites methylation level of PEG10 in CHD patients and healthy individuals

Groups	SampleID	CpG_1	CpG_2	CpG_3	CpG_4	CpG_5	CpG_6	CpG_7
Control	1	0.37	0.13	0.84	0.49	0.46	0.49	0.54
	2	0.39	0.13	1	0.49	0.52	0.49	0.54
	3	0.44	0.25	0.6	0.48	0.52	0.48	0.57
	4							
	5	0.38	0.21	0.79	0.46	0.48	0.46	0.51
	6	0.49	0.19	0.91	0.56	0.61	0.56	0.61
	7							
	8	0.52	0.14	1	0.6	0.65	0.6	0.67
	9	0.43	0.17	0.46	0.47	0.44	0.47	0.5
	10	0.39	0.19	1	0.52	0.46	0.52	0.54
	11	0.47	0.27	0.48	0.49	0.56	0.49	0.55
	12							
	13	0.44	0.21	0.33	0.46	0.52	0.46	0.55
	14	0.47	0.22	0.89	0.52	0.56	0.52	0.65
	15	0.48	0.53	0.94	0.55	0.69	0.55	0.61
	16							
	17	0.5	0.25	1	0.54	0.56	0.54	0.6
	18	0.39	0.12	0.94	0.44	0.42	0.44	0.56
	19							
	20							
	21	0.49	0.26	0.7	0.49	0.55	0.49	0.57
	22	0.46	0.36	0.54	0.54	0.55	0.54	0.62
	23	0.49	0.3	0.73	0.59	0.59	0.59	0.65
	24	0.44	0.08	1	0.59	0.57	0.59	0.74
	25	0.54	0.23	0.84	0.58	0.66	0.58	0.69
	26	0.4	0.15	0.83	0.52	0.54	0.52	0.58
	27	0.53	0.25	0.97	0.65	0.71	0.65	0.74
	28	0.54	0.29	1	0.6	0.61	0.6	0.66
CHD	1	0.41	0.31	0.4	0.51	0.54	0.51	0.52
	2	0.33	0.27	0.37	0.43	0.39	0.43	0.39
	3	0.34	0.22	0.41	0.42	0.44	0.42	0.46
	4	0.38	0.3	0.4	0.42	0.42	0.42	0.46
	5	0.35	0.3	0.51	0.44	0.42	0.44	0.48
	6	0.41	0.36	0.47	0.48	0.48	0.48	0.51
	7	0.38	0.28	0.83	0.49	0.49	0.49	0.49
	8	0.44	0.38	0.49	0.49	0.48	0.49	0.53
	9	0.37	0.29	0.6	0.5	0.53	0.5	0.51
	10							
	11							
	12	0.34	0.27	0.42	0.44	0.44	0.44	0.47
	13	0.31	0.28	0.34	0.46	0.48	0.46	0.46
	14	0.37	0.34	0.62	0.48	0.48	0.48	0.5
	15	0.46	0.38	0.52	0.51	0.52	0.51	0.53
	16	0.46	0.47	0.51	0.5	0.5	0.5	0.52
	17	0.39	0.29	0.46	0.46	0.46	0.46	0.48

18	0.34	0.2	0.51	0.45	0.45	0.45	0.47
19	0.36	0.25	0.44	0.45	0.45	0.45	0.46
20	0.35	0.34	0.62	0.5	0.49	0.5	0.48
21	0.32	0.22	0.45	0.48	0.48	0.48	0.44
22	0.56	0.54	1	0.65	0.6	0.65	0.57
23	0.39	0.31	0.51	0.48	0.5	0.48	0.52
24	0.41	0.27	0.42	0.45	0.46	0.45	0.49
25	0.39	0.26	0.38	0.44	0.46	0.44	0.48
26	0.43	0.31	0.45	0.46	0.45	0.46	0.47
27	0.4	0.29	0.37	0.43	0.43	0.43	0.45

Table S6.2 CpG sites methylation level of PEG10 in CHD patients and healthy individuals

Groups	SampleID	CpG_8	CpG_9	CpG_10.11.12
Control	1	0.49	0.51	0.2
	2	0.49	0.58	0.25
	3	0.48	0.52	0.28
	4			
	5	0.46	0.39	0.24
	6	0.56	0.5	0.27
	7			
	8	0.6	0.55	0.13
	9	0.47	0.49	0.27
	10	0.52	0.55	0.14
	11	0.49	0.61	0.41
	12			
	13	0.46	0.57	0.36
	14	0.52	0.6	0.16
	15	0.55	0.88	0.5
	16			
	17	0.54	0.7	0.26
	18	0.44	0.53	0.2
	19			
	20			
	21	0.49	0.53	0.27
	22	0.54	0.6	0.32
	23	0.59	0.67	0.37
	24	0.59	0.91	0.06
	25	0.58	0.65	0.25
	26	0.52	0.53	0.16
	27	0.65	0.77	0.15
	28	0.6	0.71	0.28
CHD	1	0.51	0.63	0.47
	2	0.43	0.45	0.37
	3	0.42	0.48	0.3
	4	0.42	0.47	0.35
	5	0.44	0.54	0.42
	6	0.48	0.55	0.45
	7	0.49	0.55	0.47
	8	0.49	0.52	0.46
	9	0.5	0.56	0.44
	10			
	11			
	12	0.44	0.46	0.34
	13	0.46	0.49	0.33
	14	0.48	0.56	0.43
	15	0.51	0.62	0.53
	16	0.5	0.55	0.5
	17	0.46	0.53	0.36

18	0.45	0.48	0.32
19	0.45	0.44	0.37
20	0.5	0.56	0.45
21	0.48	0.5	0.31
22	0.65	1	0.6
23	0.48	0.52	0.41
24	0.45	0.48	0.35
25	0.44	0.52	0.36
26	0.46	0.51	0.39
27	0.43	0.47	0.35
