# Supplementary material for: DNA methylation abnormalities of imprinted genes in congenital heart disease: a pilot study
Source: BMC Med Genomics. 2021 Jan 6;14:4. doi: 10.1186/s12920-020-00848-0 (PMC7789576; doi:10.1186/s12920-020-00848-0)
Supplement: Supplementary file 16 — Additional file 16: Table S7. CpG sites methylation level of 18 imprinted genes detected in CHD patients and healthy individuals. [file 12920_2020_848_MOESM16_ESM.pdf]

Table S7 CpG sites methylation level of MCST2 in CHD patients and healthy individuals

Groups	SampleID	CpG_1.2.3.4	CpG_5.6	CpG_7.8	CpG_9.10	CpG_11.12	CpG_13	CpG_14
Control	1	0.26	0.26	0.3	0.35	0.31	0.37	0.37
	2	0.29	0.22	0.36	0.34	0.3	0.35	0.42
	3	0.23	0.25	0.3	0.3	0.27	0.31	0.34
	4	0.32	0.29	0.34	0.33	0.3	0.36	0.44
	5	0.22	0.22	0.22	0.3	0.23	0.31	0.43
	6	0.3	0.26	0.32	0.32	0.31	0.31	0.43
	7	0.27	0.3	0.3	0.29	0.32	0.33	0.37
	8	0.27	0.27	0.29	0.3	0.28	0.32	0.39
	9							
	10	0.33	0.27	0.33	0.36	0.31	0.32	0.46
	11	0.28	0.27	0.29	0.35	0.3	0.31	0.45
	12	0.28	0.28	0.3	0.34	0.32	0.36	0.41
	13	0.21	0.24	0.24	0.36	0.23	0.3	0.4
	14	0.27	0.27	0.27	0.38	0.27	0.32	0.43
	15	0.25	0.24	0.27	0.31	0.23	0.31	0.37
	16	0.34	0.3	0.37	0.39	0.35	0.38	0.49
	17							
	18	0.32	0.24	0.33	0.36	0.28	0.36	0.48
	19	0.28	0.26	0.28	0.36	0.28	0.33	0.44
	20							
	21	0.29	0.33	0.31	0.36	0.33	0.35	0.42
	22	0.31	0.27	0.35	0.36	0.32	0.34	0.49
	23	0.27	0.28	0.31	0.33	0.27	0.31	0.41
	24	0.28	0.31	0.32	0.38	0.33	0.33	0.5
	25	0.25	0.24	0.27	0.31	0.27	0.33	0.38
	26	0.3	0.28	0.31	0.33	0.31	0.34	0.49
	27	0.31	0.32	0.34	0.38	0.36	0.38	0.49
	28	0.38	0.29	0.36	0.4	0.36	0.34	0.5
CHD	1	0.27	0.28	0.31	0.42	0.29	0.33	0.51
	2	0.3	0.28	0.32	0.36	0.3	0.37	0.41
	3	0.27	0.28	0.28	0.38	0.29	0.34	0.43
	4	0.43	0.35	0.4	0.42	0.44	0.41	0.66
	5	0.3	0.3	0.33	0.34	0.29	0.36	0.41
	6	0.33	0.29	0.32	0.35	0.34	0.39	0.39
	7	0.31	0.34	0.33	0.38	0.34	0.36	0.48
	8	0.34	0.32	0.37	0.4	0.34	0.36	0.44
	9	0.29	0.26	0.3	0.36	0.29	0.34	0.42
	10	0.3	0.3	0.31	0.37	0.33	0.37	0.54
	11	0.25	0.24	0.24	0.34	0.26	0.32	0.4
	12	0.25	0.26	0.27	0.33	0.27	0.35	0.41
	13	0.27	0.29	0.29	0.34	0.32	0.34	0.44
	14							
	15	0.3	0.32	0.3	0.3	0.33	0.37	0.47
	16	0.3	0.29	0.33	0.35	0.34	0.36	0.43
	17	0.32	0.27	0.28	0.39	0.26	0.34	0.54
	18	0.22	0.25	0.26	0.32	0.26	0.32	0.36

19	0.28	0.22	0.27	0.28	0.25	0.31	0.42
20	0.28	0.25	0.31	0.33	0.29	0.33	0.45
21	0.3	0.3	0.28	0.39	0.26	0.37	0.5
22							
23	0.29	0.27	0.28	0.3	0.33	0.33	0.4
24	0.3	0.33	0.33	0.39	0.36	0.38	0.48
25	0.24	0.24	0.3	0.35	0.3	0.33	0.42
26	0.25	0.25	0.32	0.35	0.29	0.34	0.38
27	0.24	0.23	0.27	0.3	0.26	0.31	0.4
