# Supplementary material for: DNA methylation abnormalities of imprinted genes in congenital heart disease: a pilot study
Source: BMC Med Genomics. 2021 Jan 6;14:4. doi: 10.1186/s12920-020-00848-0 (PMC7789576; doi:10.1186/s12920-020-00848-0)
Supplement: Supplementary file 17 — Additional file 17: Table S8. CpG sites methylation level of 18 imprinted genes detected in CHD patients and healthy individuals. [file 12920_2020_848_MOESM17_ESM.pdf]

Table S8 CpG sites methylation level of NNAT in CHD patients and healthy individuals

Groups	SampleID	CpG_1	CpG_2.3	CpG_4
	1	1	0.6	0.77
	2	1	0.98	0.92
	3			
	4	1	0.98	0.83
	5	0.99	0.98	0.87
	6	1	0.97	0.82
	7	0.82	0.76	0.8
	8	0.7	0.68	0.71
	9			
	10	1	0.97	0.86
	11			
	12	1	0.97	0.79
	13	0.43	0.98	0.82
Control	14	1	0.96	0.85
	15			
	16			
	17	1	0.98	0.88
	18			
	19			
	20			
	21	0.89	0.98	0.91
	22	0.96	0.98	0.92
	23	1	0.97	0.9
	24	0.72	0.21	0.82
	25	0.74	0.99	0.88
	26	0.93	0.98	0.88
	27	1	0.98	0.96
	28	1	0.99	0.9
	1	0.89	0.98	0.87
	2	1	0.98	0.85
	3	1	0.97	0.83
	4			
	5	0.83	0.6	0.82
	6	0.19	0.14	0.62
	7	1	0.98	0.89
	8	1	0.98	0.88
	9	0.86	0.98	0.91
	10	1	0.97	0.94
	11	1	0.97	0.81
	12	1	0.82	0.86
	13	1	0.98	0.86
CHD	14	0.39	0.98	0.91
	15			
	16	1	0.97	0.86
	17	1	0.97	0.84

18	1	0.97	0.91
19	0.54	0.97	0.79
20	0.8	0.97	0.83
21	1	0.97	0.89
22	0.98	0.98	0.92
23	1	0.97	0.79
24			
25	1	0.98	0.91
26	1	0.99	0.84
27	1	0.98	0.91
