# Supplementary material for: DNA methylation abnormalities of imprinted genes in congenital heart disease: a pilot study
Source: BMC Med Genomics. 2021 Jan 6;14:4. doi: 10.1186/s12920-020-00848-0 (PMC7789576; doi:10.1186/s12920-020-00848-0)
Supplement: Supplementary file 18 — Additional file 18: Table S9. CpG sites methylation level of 18 imprinted genes detected in CHD patients and healthy individuals. [file 12920_2020_848_MOESM18_ESM.pdf]

Table S9 CpG sites methylation level of NAP1L5 in CHD patients and healthy individuals

Groups	SampleID	CpG_1	CpG_2	CpG_3	CpG_4	CpG_5	CpG_6	CpG_7
	1	1	0.97	0.99	0.53	0.31	0.49	0.54
	2	1	0.96	0.9	0.55	0.31	0.5	0.55
	3	0.99	0.99	0.96	0.57	0.34	0.5	0.57
	4	1	0.94	0.89	0.54	0.28	0.5	0.54
	5	0.97	0.98	1	0.68	0.31	0.52	0.58
	6	1	0.94	0.93	0.57	0.29	0.47	0.51
	7	1	0.95	1	0.71	0.29	0.49	0.55
	8	1	0.96	0.92	0.6	0.29	0.49	0.51
	9							
	10	1	0.98	0.97	0.68	0.31	0.5	0.55
	11							
	12	1	0.98	0.88	0.58	0.32	0.44	0.54
	13	0.97	0.97	0.93	0.66	0.31	0.51	0.55
	14	0.96	0.98	1	0.72	0.24	0.45	0.33
Control	15	1	0.95	0.95	0.65	0.33	0.54	0.57
	16							
	17	0.99	0.94	0.88	0.72	0.35	0.57	0.63
	18	0.82	0.91	1	0.5	0.3	0.52	0.57
	19							
	20							
	21	0.9	0.95	0.86	0.52	0.27	0.51	0.54
	22	0.83	0.96	0.91	0.58	0.32	0.55	0.55
	23	0.83	0.96	0.96	0.65	0.3	0.56	0.61
	24	0.76	0.93	0.19	0.42	0.34	0.52	0.58
	25	1	0.91	1	1	0.29	0.51	0.57
	26	1	0.98	0.97	0.54	0.29	0.51	0.49
	27	1	0.94	0.98	0.89	0.33	0.6	0.57
	28	1	0.98	0.95	0.65	0.29	0.49	0.55
	1	1	0.98	0.87	0.64	0.36	0.55	0.59
	2	0.74	0.95	0.81	0.63	0.29	0.48	0.54
	3	0.89	0.95	0.94	0.58	0.36	0.55	0.58
	4	0.54	0.87	0.17	0.29	0.3	0.49	0.55
	5	0.38	0.96	0.82	0.29	0.34	0.53	0.56
	6							
	7	0.98	0.96	0.95	0.57	0.34	0.52	0.54
	8	0.87	0.99	0.84	0.68	0.32	0.55	0.55
	9	0.68	0.95	0.49	0.52	0.35	0.53	0.6
	10	0.27	0.92	0.47	0.27	0.33	0.52	0.54
	11							
	12							
	13	0.85	0.98	0.52	0.57	0.35	0.46	0.55
CHD	14							
	15							
	16	0.42	0.62	0.08	0.44	0.32	0.5	0.54
	17	0.71	0.69	0.07	0.61	0.29	0.47	0.5
	18	0.93	0.95	0.93	0.64	0.32	0.49	0.55

19	1	0.96	0.79	0.58	0.35	0.53	0.55
20	1	0.98	0.94	0.71	0.33	0.42	0.52
21							
22	0.32	1	0.49	0.38	0.33	0.52	0.53
23	0.77	0.92	0.9	0.86	0.39	0.64	0.59
24							
25	0.88	1	0.95	0.64	0.3	0.45	0.54
26	0.86	0.91	0.98	0.74	0.3	0.5	0.55
27	1	0.97	0.99	0.88	0.34	0.52	0.55
