# Supplementary material for: DNA methylation abnormalities of imprinted genes in congenital heart disease: a pilot study
Source: BMC Med Genomics. 2021 Jan 6;14:4. doi: 10.1186/s12920-020-00848-0 (PMC7789576; doi:10.1186/s12920-020-00848-0)
Supplement: Supplementary file 19 — Additional file 19: Table S10. CpG sites methylation level of 18 imprinted genes detected in CHD patients and healthy individuals. [file 12920_2020_848_MOESM19_ESM.pdf]

Table S10.1 CpG sites methylation level of NESP in CHD patients and healthy individuals

Groups	SampleID	CpG_1	CpG_2	CpG_3	CpG_4.5	CpG_6	CpG_7	CpG_8.9.10
Control	1	0.47	0.45	0.45	0.31	0.46	0.37	0.39
	2	0.43	0.45	0.42	0.37	0.47	0.39	0.48
	3	0.44	0.48	0.48	0.42	0.45	0.38	0.41
	4	0.44	0.34	0.42	0.35	0.47	0.34	0.4
	5							
	6							
	7							
	8	0.47	0.38	0.44	0.37	0.48	0.35	0.41
	9	0.47	0.42	0.38	0.29	0.42	0.38	0.43
	10	0.46	0.4	0.42	0.33	0.42	0.38	0.47
	11							
	12	0.93	0.34	0.37	0.3	0.41	0.4	0.44
	13	0.44	0.43	0.45	0.38	0.39	0.34	0.38
	14	0.83	0.45	0.45	0.39	0.45	0.38	0.44
	15	0.45	0.44	0.43	0.35	0.45	0.38	0.46
	16	0.45	0.42	0.48	0.32	0.45	0.35	0.4
	17	0.43	0.46	0.46	0.35	0.41	0.4	0.46
	18	0.45	0.57	0.54	0.47	0.46	0.41	0.46
	19	0.46	0.49	0.49	0.36	0.47	0.42	0.5
	20							
	21	0.74	0.51	0.46	0.43	0.45	0.41	0.47
	22	0.4	0.37	0.4	0.29	0.45	0.34	0.39
	23	0.52	0.5	0.52	0.44	0.4	0.4	0.46
	24	0.49	0.49	0.45	0.44	0.43	0.37	0.44
	25	0.42	0.46	0.46	0.36	0.43	0.43	0.51
	26	0.42	0.39	0.39	0.31	0.42	0.39	0.44
	27	0.38	0.41	0.39	0.31	0.45	0.47	0.52
	28	0.42	0.43	0.41	0.4	0.49	0.4	0.44
CHD	1							
	2							
	3							
	4							
	5							
	6	0.57	0.47	0.44	0.3	0.39	0.27	0.26
	7							
	8	0.52	0.37	0.43	0.3	0.41	0.27	0.28
	9							
	10							
	11							
	12							
	13	0.51	0.29	0.41	0.28	0.39	0.29	0.3
	14							
	15							
	16							

17	0.47	0.38	0.41	0.38	0.44	0.28	0.33
18	0.42	NA	0.37	0.3	0.39	0.25	0.29
19							
20	0.56	NA	0.41	0.3	0.4	0.23	0.22
21							
22	0.5	0.37	0.42	0.34	0.41	0.27	0.3
23							
24							
25							
26							
27							

Table S10.2 CpG sites methylation level of NESP in CHD patients and healthy individuals

Groups	SampleID	CpG_11.1	CpG_13	CpG_14	CpG_15	CpG_16	CpG_17.18.19.20.21.22
	1	0.36	0.43	0.31	0.36	0.38	0.3
	2	0.4	0.46	0.31	0.42	0.44	0.32
	3	0.37	0.5	0.34	0.43	0.43	0.31
	4	0.33	0.44	0.27	0.36	0.39	0.25
	5						
	6						
	7						
	8	0.33	0.46	0.24	0.34	0.36	0.31
	9	0.34	0.52	0.3	0.37	0.37	0.27
	10	0.43	0.43	0.35	0.41	0.39	0.47
	11						
	12	0.41	0.45	0.4	0.43	0.4	0.42
	13	0.32	0.44	0.25	0.31	0.31	0.27
Control	14	0.35	0.51	0.27	0.36	0.4	0.29
	15	0.37	0.55	0.3	0.38	0.4	0.31
	16	0.31	0.38	0.29	0.35	0.35	0.32
	17	0.4	0.47	0.41	0.43	0.42	0.4
	18	0.4	0.55	0.33	0.41	0.47	0.33
	19	0.43	0.5	0.46	0.46	0.42	0.46
	20						
	21	0.42	0.41	0.44	0.45	0.42	0.41
	22	0.29	0.5	0.26	0.37	0.37	0.29
	23	0.43	0.44	0.43	0.46	0.48	0.4
	24	0.39	0.56	0.3	0.39	0.45	0.35
	25	0.45	0.76	0.4	0.45	0.5	0.44
	26	0.41	0.43	0.36	0.43	0.4	0.43
	27	0.46	0.56	0.42	0.49	0.45	0.38
	28	0.39	0.57	0.38	0.39	0.42	0.39
	1						
	2						
	3						
	4						
	5						
	6	0.27	0.24	0.18	0.21	0.24	0.25
	7						
	8	0.32	0.22	0.15	NA	0.51	0.29
	9						
	10						
	11						
	12						
	13	0.29	0.29	0.22	0.22	0.24	0.22
CHD	14						
	15						
	16						

17	0.29	0.34	0.24	0.38	0.34		0.27
18	0.24	0.29	0.21	0.26	0.26		0.24
19							
20	0.19	0.27	0.18	0.25	0.24		0.17
21							
22	0.26	0.36	0.21	0.24	0.27		0.23
23							
24							
25							
26							
27							

Table S10.3 CpG sites methylation level of NESP in CHD patients and healthy individuals

Groups	SampleID	CpG_23
Control	1	0.32
	2	0.43
	3	0.38
	4	0.3
	5	
	6	
	7	
	8	0.28
	9	0.32
	10	0.45
	11	
	12	0.44
	13	0.25
	14	0.32
	15	0.34
	16	0.32
	17	0.38
	18	0.35
	19	0.45
	20	
	21	0.45
	22	0.32
	23	0.48
	24	0.32
	25	0.41
	26	0.37
	27	0.43
	28	0.32
CHD	1	
	2	
	3	
	4	
	5	
	6	0.22
	7	
	8	0.47
	9	
	10	
	11	
	12	
	13	0.22
	14	
	15	
	16	

17	0.33
18	0.25
19	
20	0.24
21	
22	0.25
23	
24	
25	
26	
27	
