# Supplementary material for: DNA methylation abnormalities of imprinted genes in congenital heart disease: a pilot study
Source: BMC Med Genomics. 2021 Jan 6;14:4. doi: 10.1186/s12920-020-00848-0 (PMC7789576; doi:10.1186/s12920-020-00848-0)
Supplement: Supplementary file 21 — Additional file 21: Table S12. CpG sites methylation level of 18 imprinted genes detected in CHD patients and healthy individuals. [file 12920_2020_848_MOESM21_ESM.pdf]

Table S12.1 CpG sites methylation level of GNAS in CHD patients and healthy individuals

Groups	SampleID	CpG_1	CpG_2	CpG_3	CpG_4.5.6	CpG_7	CpG_8	CpG_9.10
	1							
	2							
	3	0.45	0.2	0.38	0.51	0.98	0.43	0.4
	4	0.39	0.19	0.48	0.64	0.79	0.45	0.59
	5							
	6	0.49	0.2	0.44	0.54	0.63	0.45	0.39
	7	0.39	0.24	0.46	0.54	NA	0.47	0.34
	8	0.38	0.21	0.47	0.57	1	0.48	0.47
	9	0.44	0.24	0.42	0.53	NA	0.42	0.32
	10	0.39	0.3	0.39	0.55	0.71	0.44	0.43
	11							
	12	0.48	0.31	0.51	0.58	0.51	0.45	0.47
	13	0.4	0.18	0.33	0.49	0.69	0.48	0.32
Control	14	0.4	0.23	0.53	0.52	0.39	0.38	0.35
	15	0.41	0.49	0.38	0.54	NA	0.43	0.43
	16	0.44	0.3	0.45	0.61	0.65	0.48	0.47
	17	0.39	0.34	0.4	0.56	0.46	0.37	0.35
	18	0.47	0.23	0.46	0.53	0.13	0.38	0.29
	19	0.5	0.25	0.54	0.63	0.6	0.52	0.52
	20							
	21	0.38	0.21	0.44	0.46	0.63	0.45	0.37
	22	0.47	0.18	0.49	0.54	0.4	0.45	0.4
	23	0.48	0.19	0.56	0.54	1	0.45	0.47
	24	0.51	0.12	0.59	0.65	0.31	0.58	0.56
	25	0.48	0.16	0.37	0.53	0.42	0.45	0.31
	26	0.45	0.23	0.45	0.59	0.45	0.44	0.43
	27	0.46	0.11	0.56	0.59	1	0.35	0.36
	28	0.45	0.18	0.46	0.58	0.5	0.38	0.43
	1	0.4	0.25	0.44	0.51	0.47	0.36	0.31
	2							
	3	0.46	0.3	0.48	0.58	NA	0.47	0.46
	4	0.43	0.2	0.42	0.52	0.39	0.45	0.32
	5	0.44	0.22	0.45	0.52	0.45	0.43	0.36
	6	0.4	0.16	0.34	0.49	0	0.34	0.33
	7	0.5	0.24	0.47	0.58	0.31	0.47	0.47
	8	0.41	0.27	0.38	0.54	0.45	0.39	0.34
	9	0.44	0.24	0.43	0.54	0.57	0.44	0.32
	10	0.53	0.22	0.52	0.5	1	0.46	0.45
	11	0.46	0.2	0.48	0.56	0.53	0.42	0.4
	12	0.46	0.22	0.42	0.58	0.47	0.44	0.38
	13	0.41	0.28	0.49	0.58	0.37	0.57	0.42
CHD	14							
	15	0.44	0.21	0.47	0.54	0.42	0.46	0.39
	16	0.52	0.26	0.57	0.64	0.56	0.59	0.55
	17	0.46	0.1	0.5	0.44	0.86	0.41	0.37
	18	0.43	0.35	0.47	0.55	0.71	0.39	0.35

19	0.5	0.22	0.49	0.59	0.57	0.42	0.42
20	0.33	0.16	0.4	0.55	0.55	0.39	0.38
21	NA	0.07	0.52	0.57	0.53	0.4	0.44
22	0.55	0.2	0.82	0.66	1	0.41	0.5
23	0.5	0.1	0.49	0.62	0.99	0.48	0.41
24	0.46	0.11	0.44	0.51	0.57	0.28	0.4
25	0.45	0.16	0.49	0.6	0.54	0.43	0.42
26							
27	0.41	0.23	0.37	0.51	0.41	0.39	0.29

Table S12.2 CpG sites methylation level of GNAS in CHD patients and healthy individuals

Groups	SampleID	CpG_11.12	CpG_13	CpG_14	CpG_15	CpG_16.17	CpG_18	CpG_19
Control	1							
	2							
	3	0.33	0.2	0.21	0.4	0.31	0.43	0.48
	4	0.41	0.19	0.32	0.53	0.43	0.45	0.57
	5							
	6	0.47	0.2	0.25	0.49	0.29	0.45	0.59
	7	0.49	0.24	0.25	0.45	0.33	0.47	0.48
	8	0.43	0.21	0.21	0.37	0.39	0.48	0.45
	9	0.41	0.24	0.26	0.39	0.26	0.42	0.46
	10	0.36	0.3	0.3	0.55	0.49	0.44	0.44
	11							
	12	0.45	0.31	0.28	0.46	0.39	0.45	0.51
	13	0.4	0.18	0.21	0.45	0.12	0.48	0.42
	14	0.49	0.23	0.22	0.46	0.25	0.38	0.49
	15	0.37	0.49	0.26	0.45	0.3	0.43	0.48
	16	0.46	0.3	0.3	0.45	0.46	0.48	0.5
	17	0.34	0.34	0.26	0.43	0.49	0.37	0.45
	18	0.45	0.23	0.3	0.46	0.46	0.38	0.47
	19	0.55	0.25	0.33	0.53	0.53	0.52	0.58
	20							
	21	0.39	0.21	0.24	0.45	0.36	0.45	0.51
	22	0.43	0.18	0.26	0.45	0.38	0.45	0.47
	23	0.47	0.19	0.28	0.49	0.46	0.45	0.5
	24	0.49	0.12	0.32	0.61	0.49	0.58	0.65
	25	0.44	0.16	0.23	0.44	0.36	0.45	0.5
	26	0.45	0.23	0.28	0.47	0.49	0.44	0.53
	27	0.39	0.11	0.29	0.45	0.36	0.35	0.49
	28	0.4	0.18	0.27	0.41	0.49	0.38	0.46
CHD	1	0.29	0.25	0.26	0.42	0.38	0.36	0.45
	2							
	3	0.45	0.3	0.32	0.5	0.43	0.47	0.5
	4	0.44	0.2	0.24	0.47	0.29	0.45	0.5
	5	0.33	0.22	0.24	0.4	0.22	0.43	0.46
	6	0.3	0.16	0.19	0.36	0.22	0.34	0.39
	7	0.44	0.24	0.27	0.47	0.47	0.47	0.55
	8	0.28	0.27	0.26	0.42	0.38	0.39	0.43
	9	0.42	0.24	0.25	0.43	0.39	0.44	0.48
	10	0.51	0.22	0.31	0.49	0.42	0.46	0.54
	11	0.45	0.2	0.28	0.46	0.3	0.42	0.49
	12	0.45	0.22	0.31	0.44	0.36	0.44	0.47
	13	0.43	0.28	0.27	0.46	0.41	0.57	0.52
	14							
	15	0.45	0.21	0.25	0.47	0.33	0.46	0.5
	16	0.55	0.26	0.35	0.58	0.63	0.59	0.64
	17	0.39	0.1	0.28	0.45	0.1	0.41	0.48
	18	0.44	0.35	0.36	0.48	0.49	0.39	0.52

19	0.51	0.22	0.31	0.5	0.41	0.42	0.53
20	0.44	0.16	0.23	0.4	0.39	0.39	0.41
21	0.38	0.07	0.3	0.44	0.41	0.4	0.51
22	0.72	0.2	0.61	0.7	0.78	0.41	0.72
23	0.43	0.1	0.3	0.5	0.44	0.48	0.5
24	0.41	0.11	0.26	0.39	0.29	0.28	0.5
25	0.45	0.16	0.28	0.45	0.4	0.43	0.51
26							
27	0.39	0.23	0.21	0.38	0.24	0.39	0.43
