# Supplementary material for: DNA methylation abnormalities of imprinted genes in congenital heart disease: a pilot study
Source: BMC Med Genomics. 2021 Jan 6;14:4. doi: 10.1186/s12920-020-00848-0 (PMC7789576; doi:10.1186/s12920-020-00848-0)
Supplement: Supplementary file 22 — Additional file 22: Table S13. CpG sites methylation level of 18 imprinted genes detected in CHD patients and healthy individuals. [file 12920_2020_848_MOESM22_ESM.pdf]

Table S13.1 CpG sites methylation level of PLAGL1 in CHD patients and healthy individuals

Groups	SampleID	CpG_1.2	CpG_3.4.5	CpG_6.7	CpG_8	CpG_9.10.11.12.13	CpG_14	CpG_15.16
Control	1	0.38	0.36	0.42	0.36	0.56	0.31	0.16
	2							
	3	0.4	0.47	0.38	0.39	0.63	0.44	0.19
	4	0.45	0.47	0.49	0.52	0.61	0.48	0.17
	5	0.39	0.37	0.39	0.32	0.53	0.25	0.15
	6	0.43	0.41	0.33	0.41	0.53	0.32	0.12
	7	0.38	0.35	0.38	0.34	0.53	0.29	0.13
	8							
	9	0.36	0.34	0.39	0.37	0.55	0.31	0.14
	10	0.33	0.32	0.37	0.37	0.53	0.27	0.15
	11	0.41	0.42	0.38	0.39	0.58	0.28	0.14
	12	0.36	0.41	0.4	0.39	0.56	0.34	0.13
	13	0.42	0.41	0.39	0.2	0.62	0.48	0.15
	14	0.4	0.41	0.44	0.45	0.59	0.4	0.15
	15	0.39	0.35	0.39	0.36	0.57	0.32	0.16
	16	0.42	0.43	0.45	0.38	0.59	0.31	0.18
	17	0.3	0.34	0.34	0.36	0.53	0.3	0.11
	18	0.32	0.36	0.4	0.41	0.5	0.27	0.12
	19	0.33	0.34	0.38	0.35	0.5	0.29	0.12
	20							
	21	0.47	0.46	0.48	0.5	0.64	0.4	0.19
	22	0.32	0.39	0.36	0.45	0.58	0.31	0.13
	23	0.36	0.39	0.4	0.46	0.59	0.36	0.18
	24	0.42	0.43	0.45	0.39	0.54	0.4	0.18
	25	0.33	0.38	0.37	0.38	0.59	0.3	0.14
	26	0.35	0.3	0.38	0.26	0.57	0.24	0.15
	27	0.39	0.41	0.43	0.43	0.6	0.42	0.15
	28	0.27	0.3	0.34	0.41	0.48	0.26	0.11
CHD	1	0.34	0.35	0.4	0.36	0.57	0.26	0.15
	2	0.32	0.31	0.41	0.45	0.49	0.33	0.14
	3	0.41	0.38	0.38	0.37	0.6	0.32	0.13
	4	0.29	0.4	0.36	0.4	0.5	0.21	0.13
	5	0.31	0.34	0.36	0.4	0.52	0.3	0.13
	6	0.29	0.47	0.39	0.43	0.52	0.32	0.14
	7	0.33	0.35	0.35	0.42	0.53	0.28	0.12
	8	0.3	0.34	0.29	0.33	0.5	0.22	0.13
	9							
	10	0.29	0.32	0.41	0.38	0.5	0.27	0.14
	11	0.32	0.32	0.4	0.33	0.51	0.26	0.14
	12	0.3	0.33	0.39	0.42	0.49	0.3	0.14
	13	0.37	0.39	0.41	0.41	0.59	0.31	0.15
	14	0.24	0.26	0.31	0.21	0.46	0.22	0.11
	15	0.34	0.38	0.39	0.4	0.51	0.31	0.14
	16	0.31	0.34	0.39	0.36	0.5	0.29	0.14
	17	0.37	0.32	0.45	0.35	0.55	0.31	0.14

18	0.22	0.26	0.31	0.31	0.42	0.23	0.1
19	0.4	0.43	0.47	0.41	0.62	0.36	0.17
20	0.35	0.38	0.45	0.44	0.54	0.26	0.17
21	0.4	0.36	0.45	0.39	0.5	0.31	0.16
22	0.35	0.27	0.44	0.4	0.51	0.26	0.16
23	0.37	0.43	0.41	0.39	0.62	0.3	0.14
24	0.43	0.46	0.55	0.47	0.6	0.15	0.2
25	0.31	0.38	0.42	0.39	0.49	0.28	0.17
26	0.26	0.33	0.34	0.44	0.52	0.27	0.1
27	0.31	0.32	0.37	0.35	0.54	0.29	0.14

Table S13.2 CpG sites methylation level of PLAGL1 in CHD patients and healthy individuals

Groups	SampleID	CpG_17.18.19	CpG_20	CpG_21	CpG_22.23.24	CpG_25	CpG_26	CpG_27
Control	1	0.37	0.26	0.36	0.35	0.47	0.47	0.53
	2							
	3	0.47	0.45	0.39	0.34	0.47	0.56	0.53
	4	0.43	0.39	0.52	0.43	0.51	0.73	0.58
	5	0.31	0.19	0.32	0.65	0.41	0.56	0.56
	6	0.48	0.28	0.41	0.37	0.47	0.71	0.61
	7	0.36	0.17	0.34	0.31	0.44	0.57	0.57
	8							
	9	0.37	0.27	0.37	0.32	0.45	0.5	0.59
	10	0.35	0.38	0.37	0.35	0.47	0.49	0.58
	11	0.4	0.16	0.39	0.28	0.42	0.65	0.58
	12	0.35	0.39	0.39	0.32	0.47	0.69	0.57
	13	0.39	0.21	0.2	0.35	0.5	0.57	0.56
	14	0.51	0.32	0.45	0.71	0.44	0.57	0.57
	15	0.41	0.23	0.36	0.1	0.46	0.67	0.58
	16	0.49	0.32	0.38	0.49	0.53	0.64	0.57
	17	0.31	0.29	0.36	0.27	0.42	0.54	0.57
	18	0.35	0.39	0.41	0.39	0.47	0.36	0.56
	19	0.32	0.21	0.35	0.3	0.42	0.52	0.58
	20							
	21	0.42	0.42	0.5	0.37	0.53	0.55	0.58
	22	0.32	0.28	0.45	0.31	0.43	0.55	0.56
	23	0.43	0.32	0.46	0.44	0.46	0.65	0.61
	24	0.49	0.22	0.39	0.39	0.55	0.59	0.58
	25	0.38	0.2	0.38	0.36	0.44	0.56	0.55
	26	0.37	0.15	0.26	0.37	0.47	0.66	0.55
	27	0.44	0.31	0.43	0.38	0.49	0.73	0.64
	28	0.31	0.29	0.41	0.36	0.41	0.55	0.56
CHD	1	0.32	0.31	0.36	0.3	0.47	0.53	0.52
	2	0.37	0.36	0.45	0.36	0.48	0.47	0.5
	3	0.39	0.23	0.37	0.35	0.41	0.52	0.55
	4	0.36	0.3	0.4	0.41	0.45	0.53	0.49
	5	0.31	0.37	0.4	0.3	0.46	0.51	0.5
	6	0.37	0.37	0.43	0.36	0.49	0.37	0.53
	7	0.33	0.32	0.42	0.33	0.45	0.64	0.56
	8	0.31	0.25	0.33	0.32	0.42	0.56	0.51
	9							
	10	0.36	0.35	0.38	0.38	0.51	0.63	0.52
	11	0.31	0.19	0.33	0.28	0.43	0.56	0.52
	12	0.41	0.35	0.42	0.43	0.45	0.48	0.51
	13	0.38	0.31	0.41	0.38	0.45	0.66	0.52
	14	0.3	0.26	0.21	0.4	0.47	0.61	0.65
	15	0.38	0.29	0.4	0.36	0.42	0.72	0.53
	16	0.33	0.3	0.36	0.31	0.45	0.55	0.55
	17	0.4	0.31	0.35	0.41	0.45	0.49	0.51

18	0.24	0.21	0.31	0.27	0.42	0.42	0.49
19	0.4	0.39	0.41	0.33	0.46	0.48	0.5
20	0.44	0.37	0.44	0.48	0.53	0.45	0.52
21	0.33	0.23	0.39	0.37	0.49	0.65	0.59
22	0.42	0.33	0.4	0.43	0.55	0.22	0.55
23	0.38	0.3	0.39	0.39	0.42	0.69	0.52
24	0.51	0.25	0.47	0.48	0.57	0.62	0.52
25	0.41	0.36	0.39	0.48	0.48	0.44	0.54
26	0.34	0.29	0.44	0.37	0.37	0.55	0.47
27	0.33	0.23	0.35	0.3	0.4	0.54	0.51

Table S13.3 CpG sites methylation level of PLAGL1 in CHD patients and healthy individuals

Groups	SampleID	CpG_28	CpG_29	CpG_30.31
Control	1	0.65	0.51	0.42
	2			
	3	0.68	0.48	0.44
	4	0.68	0.47	0.51
	5	0.63	0.5	0.41
	6	0.68	0.55	0.44
	7	0.69	0.48	0.45
	8			
	9	0.69	0.48	0.45
	10	0.71	0.54	0.51
	11	0.65	0.56	0.38
	12	0.69	0.54	0.47
	13	0.67	0.44	0.46
	14	0.66	0.52	0.41
	15	0.68	0.47	0.46
	16	0.66	0.49	0.46
	17	0.76	0.58	0.49
	18	0.72	0.58	0.42
	19	0.69	0.47	0.46
	20			
	21	0.33	0.52	0.47
	22	0.64	0.52	0.43
	23	0.74	0.48	0.52
	24	0.67	0.52	0.42
	25	0.67	0.43	0.47
	26	0.65	0.5	0.47
	27	0.73	0.47	0.57
	28	0.66	0.48	0.46
CHD	1	0.65	0.54	0.47
	2	0.67	0.52	0.43
	3	0.62	0.58	0.48
	4	0.66	0.5	0.45
	5	0.62	0.48	0.41
	6	0.68	0.49	0.46
	7	0.67	0.57	0.46
	8	0.71	0.6	0.47
	9			
	10	0.65	0.55	0.42
	11	0.61	0.5	0.43
	12	0.64	0.53	0.4
	13	0.63	0.51	0.47
	14	0.76	0.65	0.56
	15	0.65	0.52	0.51
	16	0.67	0.56	0.51
	17	0.6	0.46	0.39

18	0.61	0.49	0.44
19	0.61	0.44	0.43
20	0.6	0.43	0.39
21	0.66	0.54	0.49
22	0.7	0.6	0.5
23	0.62	0.49	0.5
24	0.71	0.45	0.4
25	0.64	0.48	0.38
26	0.63	0.51	0.39
27	0.62	0.49	0.46
