# Supplementary material for: DNA methylation abnormalities of imprinted genes in congenital heart disease: a pilot study
Source: BMC Med Genomics. 2021 Jan 6;14:4. doi: 10.1186/s12920-020-00848-0 (PMC7789576; doi:10.1186/s12920-020-00848-0)
Supplement: Supplementary file 23 — Additional file 23: Table S14. CpG sites methylation level of 18 imprinted genes detected in CHD patients and healthy individuals. [file 12920_2020_848_MOESM23_ESM.pdf]

Table S14.1 CpG sites methylation level of MEST in CHD patients and healthy individuals

Groups	SampleID	CpG_1	CpG_2.3	CpG_4	CpG_5.6	CpG_7	CpG_8	CpG_9.10
	1							
	2	0.54	0.5	0.56	0.56	0.58	0.58	0.42
	3	0.56	0.49	0.59	0.57	0.59	0.55	0.49
	4	0.55	0.48	0.57	0.56	0.72	0.58	0.52
	5	0.54	0.49	0.56	0.58	0.64	0.62	0.52
	6	0.53	0.4	0.53	0.49	0.64	0.53	0.45
	7							
	8	0.55	0.46	0.57	0.56	0.65	0.62	0.48
	9	0.53	0.51	0.56	0.55	0.63	0.63	0.46
	10	0.52	0.49	0.55	0.54	0.57	0.59	0.46
	11							
	12	0.59	0.55	0.6	0.61	0.74	0.71	0.52
	13	0.57	0.47	0.57	0.55	0.64	0.63	0.51
Control	14	0.55	0.52	0.55	0.55	0.7	0.67	0.49
	15	0.5	0.41	0.53	0.49	0.69	0.52	0
	16	0.54	0.46	0.56	0.55	0.62	0.56	0.53
	17	0.55	0.55	0.55	0.57	0.63	0.63	0.5
	18	0.61	0.57	0.63	0.61	0.68	0.77	0.53
	19	0.6	0.58	0.65	0.61	0.56	0.65	0.59
	20							
	21	0.62	0.6	0.67	0.62	0.7	0.78	0.64
	22	0.55	0.62	0.58	0.58	0.65	0.7	0.53
	23	0.6	0.63	0.66	0.61	0.64	0.74	0.49
	24	0.56	0.5	0.57	0.54	0.68	0.57	0.51
	25	0.53	0.54	0.56	0.58	0.67	0.82	0.4
	26	0.6	0.56	0.63	0.61	0.77	0.58	0.54
	27							
	28							
	1	0.49	0.5	0.53	0.52	0.6	0.71	0.59
	2	0.59	0.53	0.65	0.6	0.66	0.58	0.57
	3							
	4	0.54	0.47	0.56	0.56	0.67	0.72	0.52
	5	0.6	0.52	0.59	0.59	0.6	0.69	0.56
	6	0.63	0.65	0.66	0.63	0.79	0.76	0.49
	7	0.51	0.46	0.57	0.53	0.65	0.6	0.6
	8	0.58	0.45	0.53	0.53	0.7	0.75	0.5
	9	0.47	0.44	0.47	0.47	0.51	0.48	0.48
	10	0.45	0.42	0.5	0.49	0.6	0.58	0.57
	11	0.53	0.48	0.58	0.56	0.63	0.55	0.54
	12	0.5	0.44	0.51	0.52	0.6	0.69	0.61
	13	0.54	0.5	0.57	0.52	0.67	0.56	0.54
CHD	14							
	15							
	16							

17							
18	0.54	0.5	0.5	0.54	0.66	0.68	0.65
19	0.57	0.55	0.58	0.57	0.62	0.76	0.56
20	0.53	0.48	0.57	0.54	0.55	0.61	0.58
21	0.68	0.5	0.59	0.53	0.56	0.66	0.8
22							
23	0.48	0.36	0.48	0.41	0.59	0.52	0.57
24	0.56	0.52	0.61	0.6	0.66	0.54	0.51
25	0.44	0.43	0.48	0.5	0.53	0.47	0.55
26	0.75	0.73	0.77	0.73	0.85	0.9	0.48
27							

Table S14.2 CpG sites methylation level of MEST in CHD patients and healthy individuals

Groups	SampleID	CpG_11.12.13.14	CpG_15.16	CpG_17	CpG_18	CpG_19.20	CpG_21.22
Control	1						
	2	0.07	0.4	0.41	0.46	0.44	0.42
	3	0.42	0.46	0.46	0.51	0.48	0.48
	4	0.46	0.51	0.5	0.54	0.52	0.53
	5	0.41	0.51	0.47	0.47	0.46	0.46
	6	0.42	0.46	0.47	0.51	0.49	0.5
	7						
	8	0.45	0.48	0.48	0.51	0.46	0.5
	9	0.38	0.42	0.41	0.4	0.4	0.46
	10	0.37	0.44	0.46	0.48	0.47	0.46
	11						
	12	0.45	0.5	0.54	0.52	0.51	0.5
	13	0.47	0.74	0.47	0.52	0.51	0.52
	14	0.46	0.81	0.5	0.52	0.51	0.55
	15	0.09	0.52	0.55	0.55	0.44	0.48
	16	0.46	0.51	0.49	0.51	0.51	0.51
	17	0.44	0.49	0.45	0.49	0.48	0.5
	18	0.47	0.61	0.57	0.57	0.56	0.55
	19	0.97	0.57	0.53	0.56	0.58	0.56
	20						
	21	0.6	0.59	0.63	0.66	0.63	0.65
	22	0.42	0.39	0.5	0.55	0.52	0.52
	23	0.45	0.46	0.5	0.53	0.49	0.52
	24	0.51	0.55	0.51	0.58	0.55	0.58
	25	0.15	0.47	0.44	0.5	0.46	0.49
	26	0.06	0.57	0.5	0.53	0.5	0.51
	27						
	28						
CHD	1	0.9	0.65	0.55	0.54	0.53	0.66
	2	0.96	0.44	0.56	0.55	0.54	0.57
	3						
	4	0.48	0.53	0.51	0.57	0.54	0.54
	5	0.51	0.53	0.58	0.6	0.57	0.59
	6	0.54	0.4	0.53	0.58	0.54	0.59
	7	0.55	0.58	0.63	0.63	0.63	0.62
	8	0.44	0.52	0.49	0.52	0.51	0.51
	9	0.42	0.47	0.48	0.48	0.48	0.53
	10	0.55	0.6	0.56	0.59	0.6	0.62
	11	0.47	0.49	0.5	0.53	0.51	0.53
	12	0.62	0.59	0.6	0.65	0.62	0.62
	13	0.48	0.55	0.55	0.56	0.54	0.56
	14						
	15						
	16						

17						
18	0.62	0.65	0.63	0.62	0.65	0.69
19	0.48	0.48	0.55	0.55	0.53	0.57
20	0.52	0.46	0.57	0.57	0.59	0.57
21	0.8	0.8	0.75	0.67	0.82	0.82
22						
23	0.47	0.5	0.52	0.57	0.58	0.58
24	0.43	0.46	0.5	0.55	0.55	0.61
25	0.48	0.52	0.52	0.51	0.55	0.57
26	0.41	0.34	0.42	0.39	0.45	0.43
27						

Table S14.3 CpG sites methylation level of MEST in CHD patients and healthy individuals

Groups	SampleID	CpG_23.24.25.26
Control	1	
	2	0.44
	3	0.46
	4	0.51
	5	0.44
	6	0.49
	7	
	8	0.46
	9	0.46
	10	0.47
	11	
	12	0.51
	13	0.51
	14	0.53
	15	0.37
	16	0.51
	17	0.53
	18	0.55
	19	0.55
	20	
	21	0.65
	22	0.48
	23	0.48
	24	0.57
	25	0.5
	26	0.51
	27	
	28	
CHD	1	0.56
	2	0.54
	3	
	4	0.53
	5	0.6
	6	0.6
	7	0.57
	8	0.5
	9	0.51
	10	0.6
	11	0.57
	12	0.63
	13	0.57
	14	
	15	
	16	

17	
18	0.73
19	0.58
20	0.59
21	0.79
22	
23	0.61
24	0.48
25	0.57
26	0.39
27	
