# Supplementary material for: DNA methylation abnormalities of imprinted genes in congenital heart disease: a pilot study
Source: BMC Med Genomics. 2021 Jan 6;14:4. doi: 10.1186/s12920-020-00848-0 (PMC7789576; doi:10.1186/s12920-020-00848-0)
Supplement: Supplementary file 24 — Additional file 24: Table S15. CpG sites methylation level of 18 imprinted genes detected in CHD patients and healthy individuals. [file 12920_2020_848_MOESM24_ESM.pdf]

Table S15.1 CpG sites methylation level of IGF2 in CHD patients and healthy individuals

Groups	SampleID	CpG_1	CpG_2	CpG_3	CpG_4.5	CpG_6	CpG_7	CpG_8
	1	0.3	0.63	0.62	0.47	0.62	0.36	0.7
	2	0.5	0.73	0.6	0.49	0.6	0.54	0.76
	3							
	4							
	5	0.41	0.66	0.58	0.58	0.58	0.36	0.78
	6							
	7	0.55	0.53	0.57	0.49	0.57	0.4	0.96
	8	1	0.5	0.64	0.41	0.64	0.38	0.43
	9	0.41	0.75	0.59	0.38	0.59	0.54	0.71
	10							
	11							
	12	0.44	0.45	0.59	0.5	0.59	0.37	0.78
	13							
Control	14	0.46	1	0.54	0.61	0.54	0.54	0.52
	15							
	16	0.41	0.39	0.58	0.48	0.58	0.47	0.86
	17	0.56	0.84	0.68	0.65	0.68	0.89	0.71
	18	0.5	0.78	0.64	0.57	0.64	0.37	0.68
	19							
	20							
	21	0.4	0.77	0.59	0.53	0.59	0.39	0.87
	22							
	23	0.5	0.73	0.64	0.51	0.64	0.49	0.8
	24	0.36	0.69	0.59	0.55	0.59	0.33	0.69
	25	0.29	1	0.56	0.54	0.56	0.4	0.72
	26	0.48	0.71	0.61	0.61	0.61	0.52	0.78
	27	0.36	0.93	0.63	0.9	0.63	0.64	0.65
	28							
	1	0.6	0.76	0.66	0.58	0.66	0.53	0.77
	2	0.49	0.73	0.61	0.58	0.61	0.45	0.66
	3							
	4	0.51	0.75	0.6	0.6	0.6	0.47	0.75
	5	0.52	0.74	0.63	0.55	0.63	0.46	0.77
	6	0.41	0.7	0.59	0.68	0.59	0.38	0.71
	7	0.3	0.68	0.56	0.44	0.56	0.38	0.66
	8	0.28	0.58	0.53	0.62	0.53	0.34	0.43
	9	0.65	0.87	0.66	0.63	0.66	0.69	0.84
	10							
	11							
	12	0.43	0.73	0.65	0.54	0.65	0.45	0.71
	13	0.49	0.63	0.6	0.52	0.6	0.98	0.69
CHD	14	0.34	0.82	0.57	0.49	0.57	0.36	0.69
	15	0.49	0.76	0.64	0.53	0.64	0.45	0.74
	16	0.43	0.7	0.55	0.52	0.55	0.41	0.74
	17	0.27	0.63	0.54	0.55	0.54	0.24	0.54
	18	0.39	0.86	0.58	0.49	0.58	0.39	0.75

19	0.51	0.9	0.68	0.62	0.68	0.59	0.77
20							
21							
22							
23	0.41	0.83	0.67	0.49	0.67	0.43	0.63
24							
25	0.12	0.66	0.78	1	0.78	0.12	0.12
26							
27	0.51	0.72	0.59	0.58	0.59	0.46	0.72

Table S15.2 CpG sites methylation level of IGF2 in CHD patients and healthy individuals

Groups	SampleID	CpG_9.10.11	CpG_12.13	CpG_14	CpG_15.16	CpG_17	CpG_18	CpG_19
Control	1	0.55	0.83	0.69	0.64	0.8	0.68	0.43
	2	0.46	0.53	0.48	0.49	0.62	0.61	0.46
	3							
	4							
	5	0.63	0.54	0.77	0.72	0.83	0.73	0.53
	6							
	7	0.47	0.52	0.47	0.62	0.67	0.58	0.55
	8	0.43	0.56	0.47	0.52	0.7	0.62	0.58
	9	0.64	0.7	0.69	0.6	0.82	0.69	0.41
	10							
	11							
	12	0.48	0.61	0.53	0.63	0.72	0.7	0.33
	13							
	14	0.45	0.61	0.6	0.59	0.74	0.69	0.44
	15							
	16	0.51	0.53	0.61	0.58	0.71	0.68	1
	17	0.46	0.52	0.51	0.63	0.74	0.7	0.42
	18	0.54	0.6	0.61	0.63	0.77	0.71	0.42
	19							
	20							
	21	0.48	0.59	0.58	0.63	0.77	0.72	0.55
	22							
	23	0.44	0.47	0.47	0.53	0.62	0.62	0.38
	24	0.53	0.71	0.58	0.67	0.74	0.73	0.46
	25	0.41	0.5	0.41	0.59	0.56	0.61	0.4
	26	0.44	0.46	0.37	0.56	0.52	0.57	0.43
	27	0.52	0.56	0.57	0.65	0.74	0.69	0.48
	28							
CHD	1	0.62	0.76	0.65	0.65	0.83	0.7	0.46
	2	0.57	0.54	0.55	0.65	0.67	0.68	0.37
	3							
	4	0.5	0.54	0.53	0.62	0.72	0.66	0.36
	5	0.48	0.45	0.49	0.56	0.73	0.63	0.5
	6	0.43	0.47	0.49	0.51	0.69	0.6	0.52
	7	0.46	0.47	0.47	0.53	0.64	0.6	0.52
	8	0.55	0.7	0.6	0.64	0.77	0.7	0.52
	9	0.57	0.61	0.59	0.66	0.8	0.69	0.59
	10							
	11							
	12	0.4	0.48	0.54	0.53	0.61	0.63	0.61
	13	0.42	0.49	0.53	0.46	0.64	0.54	0.45
	14	0.62	0.66	0.63	0.69	0.81	0.72	0.42
	15	0.46	0.55	0.57	0.57	0.73	0.65	0.51
	16	0.48	0.52	0.51	0.57	0.68	0.61	0.5
	17	0.58	0.6	0.56	0.69	0.8	0.68	0.37
	18	0.5	0.51	0.54	0.58	0.7	0.64	0.51

19	0.6	0.78	0.62	0.66	0.76	0.7	0.49
20							
21							
22							
23	0.4	0.43	0.53	0.53	0.6	0.56	0.37
24							
25	0.5	0.58	0.43	0.57	0.73	0.62	0.3
26							
27	0.62	0.66	0.66	0.73	0.81	0.77	0.37

Table S15.3 CpG sites methylation level of IGF2 in CHD patients and healthy individuals

Groups	SampleID	CpG_20	CpG_21.22	CpG_23	CpG_24.25	CpG_26	CpG_27
Control	1	0.44	0.46	0.64	0.66	0.64	0.69
	2	0.39	0.4	0.55	0.5	0.55	0.59
	3						
	4						
	5	0.43	0.53	0.66	0.63	0.66	0.72
	6						
	7	0.43	0.47	0.64	0.63	0.64	0.72
	8	0.36	0.51	0.62	0.64	0.62	0.7
	9	0.38	0.41	0.64	0.63	0.64	0.75
	10						
	11						
	12	0.36	0.39	0.61	0.6	0.61	0.69
	13						
	14	0.37	0.44	0.62	0.65	0.62	0.71
	15						
	16	0.43	0.56	0.68	0.61	0.68	0.73
	17	0.44	0.43	0.62	0.62	0.62	0.7
	18	0.46	0.5	0.65	0.69	0.65	0.64
	19						
	20						
	21	0.45	0.56	0.68	0.79	0.68	0.74
	22						
	23	0.44	0.38	0.63	0.64	0.63	0.73
	24	0.4	0.43	0.62	0.65	0.62	0.71
	25	0.38	0.36	0.58	0.58	0.58	0.62
	26	0.36	0.39	0.54	0.53	0.54	0.65
	27	0.41	0.57	0.58	0.6	0.58	0.65
	28						
CHD	1	0.51	0.6	0.73	0.76	0.73	0.8
	2	0.43	0.51	0.64	0.66	0.64	0.71
	3						
	4	0.46	0.34	0.56	0.53	0.56	0.64
	5	0.42	0.54	0.65	0.68	0.65	0.75
	6	0.36	0.47	0.55	0.55	0.55	0.6
	7	0.43	0.55	0.61	0.66	0.61	0.67
	8	0.5	0.48	0.68	0.68	0.68	0.74
	9	0.5	0.63	0.69	0.72	0.69	0.77
	10						
	11						
	12	0.48	0.6	0.66	0.69	0.66	0.77
	13	0.39	0.48	0.64	0.66	0.64	0.68
	14	0.38	0.43	0.61	0.57	0.61	0.69
	15	0.45	0.42	0.62	0.6	0.62	0.69
	16	0.39	0.5	0.63	0.7	0.63	0.67
	17	0.34	0.45	0.61	0.64	0.61	0.6
	18	0.44	0.5	0.59	0.59	0.59	0.63

19	0.39	0.45	0.68	0.58	0.68	0.73
20						
21						
22						
23	0.43	0.46	0.6	0.55	0.6	0.63
24						
25	0.35	0.41	0.59	0.55	0.59	0.7
26						
27	0.36	0.49	0.59	0.6	0.59	0.62
