# Supplementary material for: DNA methylation abnormalities of imprinted genes in congenital heart disease: a pilot study
Source: BMC Med Genomics. 2021 Jan 6;14:4. doi: 10.1186/s12920-020-00848-0 (PMC7789576; doi:10.1186/s12920-020-00848-0)
Supplement: Supplementary file 25 — Additional file 25: Table S16. CpG sites methylation level of 18 imprinted genes detected in CHD patients and healthy individuals. [file 12920_2020_848_MOESM25_ESM.pdf]

Table S16.1 CpG sites methylation level of H19 in CHD patients and healthy individuals

Groups	SampleID	CpG_1	CpG_2	CpG_3.4	CpG_5	CpG_6	CpG_7.8.9	CpG_10
	1							
	2	0.26	0.07	0.48	0.18	0	0.17	0.71
	3	0.34	0.44	0.57	0.28	0.38	0.44	0.94
	4							
	5	0.52	0.38	0.47	0.43	0.46	0.53	0.56
	6	0.53	0.59	0.6	1	0.49	0.55	0.69
	7	0.29	0.5	0.47	0.42	0.5	0.56	0.48
	8							
	9	0.38	0.39	0.36	0.4	0.4	0.43	0.65
	10	0.41	0.34	0.41	0.4	0.43	0.53	0.83
	11	0.47	0.37	0.45	0.36	0.41	0.51	0.82
	12	0.5	0.26	NA	0.51	0.28	0.41	0.66
	13	0.48	0.41	0.48	0.37	0.35	0.46	0.83
Control	14							
	15	0.57	0.27	0.43	0.31	0.4	0.44	0.68
	16							
	17	0.41	0.43	0.42	0.4	0.38	0.51	0.67
	18	0.44	0.34	0.46	0.39	0.34	0.44	0.54
	19							
	20	0.3	0.25	0.44	0.38	0.28	0.36	0.89
	21							
	22	0.41	0.33	0.33	0.31	0.33	0.46	0.63
	23							
	24	0.46	0.41	0.34	0.42	0.36	0.43	0.63
	25	0.56	NA	0.7	0.46	0.56	0.34	0.71
	26	0.55	0.34	0.43	0.43	0.35	0.31	0.82
	27	0.43	0.5	0.51	0.58	0.53	0.39	0.57
	28	0.45	NA	NA	0.53	0.43	0.22	0.18
	1	0.54	0.41	0.44	0.44	0.45	0.52	0.63
	2	0.39	0.31	0.38	0.38	0.41	0.47	0.37
	3	0.46	0.41	0.55	0.44	0.44	0.53	0.7
	4	0.56	0.34	0.35	0.4	0.3	0.36	0.4
	5	0.42	0.37	0.38	0.33	0.41	0.47	0.55
	6	0.5	0.48	0.46	0.42	0.41	0.58	0.16
	7							
	8	0.39	0.27	0.21	0.22	0.35	0.18	0.76
	9							
	10							
	11	0.52	NA	0.33	0.3	0.26	0.27	0.65
	12	0.4	0.3	0.39	0.25	0.38	0.47	0.95
	13	0.56	0.48	0.48	0.43	0.41	0.57	0.59
CHD	14							
	15	0.51	0.22	0.29	0.31	0.31	0.19	0.97
	16							
	17	0.39	0.37	0.43	0.38	0.38	0.51	0.49

18							
19							
20							
21	0.43	0.06	0.1		0.09	0.17	0.68
22							
23							
24							
25							
26							
27	0.53	0.5	0.48	0.5	0.49	0.55	0.65

Table S16.2 CpG sites methylation level of H19 in CHD patients and healthy individuals

Groups	SampleID	CpG_11	CpG_12	CpG_13.14	CpG_15	CpG_16
	1					
	2	0.21	0.75	0.06	0.24	0.8
	3	0.29	0.6	0.38	0.21	0.5
	4					
	5	0.57	0.62	0.51	0.31	0.59
	6	0.49	1	0.37	0.41	0.15
	7	0.6	0.68	0.4	0.34	0.59
	8					
	9	0.48	0.63	0.42	0.27	0.68
	10	0.53	0.67	0.44	0.32	0.6
	11	0.56	0.65	0.43	0.28	0.52
	12	0.56	0.87	0.42	0.34	0.4
	13	0.5	0.81	0.4	0.31	0.7
Control	14					
	15	0.52	0.66	0.41	0.27	0.7
	16					
	17	0.4	0.55	0.34	0.26	0.56
	18	0.58	0.82	0.41	0.26	0.27
	19					
	20	0.4	0.85	0.3	0.2	0.73
	21					
	22	0.47	0.5	0.38	0.26	0.45
	23					
	24	0.36	0.63	0.34	0.25	0.78
	25	0.53	NA	0.77	0.44	0.58
	26	0.27	0.82	0.31	0.33	0.43
	27	0.5	0.6	0.43	0.41	0.59
	28	0.36	1	0.44	0.4	0.75
	1	0.78	0.73	0.44	0.29	0.31
	2	0.4	0.65	0.37	0.28	0.44
	3	0.51	0.79	0.48	0.3	0.7
	4	0.77	0.6	0.36	0.21	0.55
	5	0.43	0.57	0.32	0.26	0.51
	6	0.56	0.64	0.47	0.33	0.4
	7					
	8	0.23	0.81	0.18	0.26	0.61
	9					
	10					
	11	0.61	1	0.43	0.41	0.54
	12	0.46	0.75	0.34	0.26	0.55
	13	0.5	0.6	0.46	0.31	0.49
CHD	14					
	15	0.35	0.69	0.34	0.21	0.42
	16					
	17	0.37	0.56	0.33	0.24	0.6

18					
19					
20					
21	0.24	0.75	0.13	0.23	0.85
22					
23					
24					
25					
26					
27	0.63	0.5	0.53	0.33	0.5
