# Supplementary material for: DNA methylation abnormalities of imprinted genes in congenital heart disease: a pilot study
Source: BMC Med Genomics. 2021 Jan 6;14:4. doi: 10.1186/s12920-020-00848-0 (PMC7789576; doi:10.1186/s12920-020-00848-0)
Supplement: Supplementary file 26 — Additional file 26: Table S17. CpG sites methylation level of 18 imprinted genes detected in CHD patients and healthy individuals. [file 12920_2020_848_MOESM26_ESM.pdf]

Table S17.1 CpG sites methylation level of IG in CHD patients and healthy individuals

Groups	SampleID	CpG_1.2.3	CpG_4.5	CpG_6	CpG_7	CpG_8	CpG_9.10
Control	1						
	2	0.67	0.13	0.72	0.41	0.77	0.58
	3						
	4	0.72	0.51	0.59	0.59	0.8	0.58
	5	0.62	0.2	0.57	0.56	0.69	0.61
	6	0.6	0.2	0.59	0.33	0.75	0.58
	7	0.63	0.08	0.64	0.37	0.77	0.62
	8						
	9						
	10	0.59	0.66	0.6	0.49	0.71	0.61
	11						
	12	0.63	0.55	0.51	0.7	0.74	0.54
	13	0.65	0.31	0.62	0.48	0.79	0.62
	14						
	15						
	16						
	17	0.61	0.55	0.6	0.31	0.75	0.59
	18	0.62	0.46	0.59	0.61	0.74	0.58
	19	0.54	0.48	0.58	0.43	0.79	0.59
	20						
	21	0.65	0.55	0.57	0.46	0.75	0.57
	22	0.63	0.61	0.56	0.34	0.8	0.61
	23	0.59	0.02	0.44	0.05	0.59	0.58
	24	0.68	0.03	0.57	0.07	0.54	0.61
	25	0.52	0.3	0.54	0.25	0.71	0.57
	26	0.61	0.04	0.57	0.17	0.76	0.57
	27						
	28	0.59	0.04	0.58	0.18	0.79	0.57
CHD	1	0.67	0.5	0.56	0.61	0.73	0.56
	2	0.55	0.49	0.58	0.29	0.72	0.56
	3	0.46	0.41	0.53	0.47	0.74	0.6
	4	0.55	0.48	0.55	0.69	0.73	0.54
	5	0.6	0.49	0.59	0.6	0.78	0.61
	6	0.62	0.58	0.56	0.3	0.78	0.56
	7	0.56	0.51	0.51	0.52	0.74	0.57
	8	0.61	0.55	0.54	0.16	0.69	0.55
	9	0.58	0.51	0.6	0.52	0.66	0.58
	10	0.54	0.42	0.53	0.32	0.6	0.55
	11	0.53	0.49	0.55	0.61	0.72	0.58
	12	0.58	0.52	0.52	0.3	0.75	0.6
	13						
	14						
	15	0.6	0.56	0.57	0.58	0.81	0.55
	16						
	17	0.63	0.15	0.66	0.42	0.72	0.64
	18						

19	0.51	0.47	0.45	0.46	0.66	0.61
20	0.45	0.43	0.42	0.35	0.62	0.6
21	0.37	0.02	0.51	0.06	0.51	0.53
22						
23						
24						
25	0.48	0.08	0.54	0.13	0.68	0.64
26						
27						

Table S17.2 CpG sites methylation level of IG in CHD patients and healthy individuals

Groups	SampleID	CpG_11.12	CpG_13	CpG_14	CpG_15	CpG_16	CpG_17	CpG_18
Control	1							
	2	0.63	0.66	0.41	0.41	0.42	0.52	0.62
	3							
	4	0.7	0.76	0.44	0.44	0.5	0.46	0.53
	5	0.68	0.7	0.45	0.45	0.48	0.4	0.46
	6	0.66	0.66	0.42	0.42	0.48	0.37	0.42
	7	0.67	0.68	0.46	0.46	0.48	0.45	0.44
	8							
	9							
	10	0.76	0.72	0.45	0.45	0.51	0.4	0.44
	11							
	12	0.61	0.67	0.38	0.38	0.4	0.35	0.55
	13	0.72	0.7	0.47	0.47	0.53	0.42	0.4
	14							
	15							
	16							
	17	0.69	0.71	0.44	0.44	0.53	0.35	0.39
	18	0.68	0.67	0.45	0.45	0.45	0.45	0.48
	19	0.67	0.68	0.43	0.43	0.47	0.37	0.41
	20							
	21	0.64	0.7	0.42	0.42	0.47	0.4	0.4
	22	0.79	0.81	0.49	0.49	0.45	0.57	0.5
	23	0.68	0.69	0.43	0.43	0.45	0.4	0.41
	24	0.72	0.72	0.46	0.46	0.56	0.48	0.39
	25	0.6	0.56	0.42	0.42	0.4	0.43	0.34
	26	0.56	0.55	0.38	0.38	0.39	0.39	0.55
	27							
	28	0.59	0.6	0.39	0.39	0.4	0.3	0.44
CHD	1	0.56	0.58	0.4	0.4	0.42	0.33	0.4
	2	0.49	0.53	0.41	0.41	0.4	0.37	0.31
	3	0.74	0.73	0.48	0.48	0.44	0.62	0.59
	4	0.6	0.6	0.39	0.39	0.39	0.42	0.45
	5	0.74	0.67	0.45	0.45	0.53	0.38	0.43
	6	0.57	0.55	0.41	0.41	0.43	0.38	0.35
	7	0.67	0.63	0.43	0.43	0.52	0.38	0.39
	8	0.63	0.61	0.41	0.41	0.45	0.4	0.4
	9	0.59	0.56	0.43	0.43	0.47	0.51	0.43
	10	0.6	0.59	0.4	0.4	0.4	0.36	0.35
	11	0.65	0.6	0.44	0.44	0.43	0.47	0.53
	12	0.7	0.6	0.43	0.43	0.37	0.4	0.43
	13							
	14							
	15	0.59	0.61	0.4	0.4	0.43	0.31	0.43
	16							
	17	0.73	0.67	0.49	0.49	0.51	0.46	0.37
	18							

19	0.7	0.7	0.45	0.45	0.49	0.39	0.42
20	0.66	0.63	0.46	0.46	0.46	0.45	0.6
21	0.53	0.48	0.36	0.36	0.34	0.39	0.27
22							
23							
24							
25	0.65	0.75	0.48	0.48	0.47	0.4	0.24
26							
27							

Table S17.3 CpG sites methylation level of IG in CHD patients and healthy individuals

Groups	SampleID	CpG_19.20	CpG_21	CpG_22	CpG_23	CpG_24	CpG_25	CpG_26
Control	1							
	2	0.64	0.6	0.86	0.68	0.57	0.55	0.55
	3							
	4	0.67	0.55	0.64	0.67	0.53	0.53	0.53
	5	0.66	0.54	0.57	0.7	0.48	0.52	0.52
	6	0.59	0.43	0.56	0.67	0.47	0.47	0.47
	7	0.69	0.53	0.7	0.7	0.52	0.5	0.5
	8							
	9							
	10	0.69	0.47	0.65	0.69	0.53	0.49	0.49
	11							
	12	0.64	0.58	0.64	0.63	0.49	0.5	0.5
	13	0.62	0.49	0.55	0.63	0.47	0.43	0.43
	14							
	15							
	16							
	17	0.61	0.48	0.62	0.65	0.44	0.49	0.49
	18	0.68	0.44	0.68	0.7	0.52	0.55	0.55
	19	0.61	0.49	0.65	0.66	0.45	0.46	0.46
	20							
	21	0.66	0.46	0.63	0.64	0.47	0.52	0.52
	22	0.79	0.56	0.77	0.81	0.6	0.57	0.57
	23	0.62	0.5	0.62	0.69	0.42	0.51	0.51
	24	0.62	0.46	0.6	0.67	0.47	0.47	0.47
	25	0.53	0.42	0.68	0.77	0.49	0.52	0.52
	26	0.6	0.46	0.61	0.64	0.49	0.56	0.56
	27							
	28	0.46	0.47	0.53	0.81	0.43	0.44	0.44
CHD	1	0.51	0.37	0.46	0.51	0.47	0.47	0.47
	2	0.57	0.38	0.56	0.6	0.45	0.49	0.49
	3	0.72	0.72	0.75	0.81	0.54	0.64	0.64
	4	0.61	0.46	0.6	0.62	0.51	0.58	0.58
	5	0.58	0.47	0.59	0.58	0.5	0.46	0.46
	6	0.53	0.35	0.5	0.56	0.5	0.45	0.45
	7	0.57	0.44	0.54	0.55	0.51	0.41	0.41
	8	0.5	0.47	0.49	0.54	0.42	0.42	0.42
	9	0.67	0.4	0.73	0.76	0.62	0.62	0.62
	10	0.54	0.44	0.57	0.72	0.43	0.42	0.42
	11	0.64	0.56	0.71	0.63	0.53	0.6	0.6
	12	0.62	0.45	0.57	0.62	0.52	0.49	0.49
	13							
	14							
	15	0.54	0.46	0.55	0.55	0.44	0.45	0.45
	16							
	17	0.57	0.41	0.58	0.62	0.48	0.48	0.48
	18							

19	0.56	0.47	0.6	0.62	0.5	0.51	0.51
20	0.62	0.61	0.68	0.67	0.53	0.54	0.54
21	0.59	0.31	0.62	0.72	0.47	0.53	0.53
22							
23							
24							
25	0.53	0.2	0.64	0.72	0.48	0.5	0.5
26							
27							
