# Supplementary material for: DNA methylation abnormalities of imprinted genes in congenital heart disease: a pilot study
Source: BMC Med Genomics. 2021 Jan 6;14:4. doi: 10.1186/s12920-020-00848-0 (PMC7789576; doi:10.1186/s12920-020-00848-0)
Supplement: Supplementary file 27 — Additional file 27: Table S18. CpG sites methylation level of 18 imprinted genes detected in CHD patients and healthy individuals. [file 12920_2020_848_MOESM27_ESM.pdf]

Table S18 CpG sites methylation level of MEG3 in CHD patients and healthy individuals

Groups	SampleID	CpG_1	CpG_2	CpG_3.4	CpG_5	CpG_6	CpG_7
	1						
	2	0.46	0.49	0.57	0.57	0.5	0.58
	3	0.41	0.41	0.33	0.45	0.38	0.52
	4	0.46	0.48	0.5	0.53	0.48	0.54
	5	0.47	0.43	0.37	0.46	0.4	0.54
	6	0.51	0.46	0.38	0.47	0.37	0.61
	7	0.48	0.49	0.43	0.54	0.42	0.61
	8	0.48	0.54	0.43	0.56	0.45	0.61
	9	0.41	0.44	0.37	0.42	0.37	0.51
	10	0.38	0.46	0.33	0.46	0.39	0.5
	11						
	12	0.4	0.4	0.33	0.4	0.34	0.47
	13	0.42	0.39	0.36	0.42	0.36	0.5
Control	14	0.48	0.47	0.5	0.51	0.41	0.59
	15	0.49	0.44	0.43	0.48	0.42	0.56
	16	0.48	0.46	0.38	0.49	0.4	0.56
	17	0.41	0.4	0.34	0.4	0.34	0.47
	18	0.42	0.4	0.37	0.46	0.35	0.51
	19	0.42	0.47	0.37	0.48	0.43	0.55
	20						
	21	0.5	0.47	0.48	0.5	0.47	0.57
	22	0.45	0.47	0.46	0.51	0.43	0.56
	23	0.42	0.41	0.32	0.42	0.37	0.49
	24	0.46	0.44	0.37	0.45	0.39	0.54
	25	0.41	0.46	0.44	0.47	0.4	0.56
	26	0.41	0.46	0.44	0.47	0.42	0.49
	27	0.48	0.46	0.34	0.47	0.4	0.58
	28						
	1						
	2	0.44	0.48	0.49	0.49	0.42	0.51
	3	0.4	0.42	0.35	0.42	0.36	0.47
	4	0.37	0.39	0.37	0.4	0.44	0.47
	5	0.37	0.35	0.28	0.4	0.33	0.46
	6	0.38	0.36	0.31	0.4	0.36	0.46
	7	0.39	0.35	NA	0.43	0.39	0.54
	8						
	9	0.37	0.37	0.31	0.35	0.28	0.41
	10	0.45	0.41	0.48	0.43	0.38	0.54
	11	0.35	0.39	0.35	0.42	0.37	0.48
	12	0.36	0.38	0.32	0.41	0.3	0.46
	13	0.36	0.37	0.29	0.39	0.34	0.44
CHD	14	0.39	0.44	0.35	0.37	0.36	0.5
	15	0.36	0.37	0.26	0.38	0.32	0.47
	16						
	17						
	18	0.36	0.44	0.41	0.43	0.33	0.53

19	0.38	0.42	0.43	0.45	0.44	0.56
20						
21						
22	0.4	0.37	0.33	0.41	0.36	0.47
23	0.39	0.39	0.32	0.37	0.32	0.45
24						
25	0.39	0.4	0.3	0.45	0.38	0.45
26	0.46	0.43	0.32	0.42	0.37	0.45
27	0.34	0.36	0.27	0.38	0.3	0.41
