# Supplementary material for: DNA methylation abnormalities of imprinted genes in congenital heart disease: a pilot study
Source: BMC Med Genomics. 2021 Jan 6;14:4. doi: 10.1186/s12920-020-00848-0 (PMC7789576; doi:10.1186/s12920-020-00848-0)
Supplement: Supplementary file 28 — Additional file 28: Table S19. CpG sites methylation level of 18 imprinted genes detected in CHD patients and healthy individuals. [file 12920_2020_848_MOESM28_ESM.pdf]

Table S19 CpG sites methylation level of KCNQ1OT1 in CHD patients and healthy individuals

Groups	SampleID	CpG_1.2.3	CpG_4.5.6	CpG_7	CpG_8.9	CpG_10.11
Control	1	NA	0.54	0.28	0.37	0.37
	2	0.37	0.5	0.27	0.38	0.38
	3	0.37	0.43	0.25	0.36	0.36
	4	0.39	0.48	0.23	0.43	0.43
	5	0.34	0.53	0.25	0.4	0.4
	6	0.38	0.4	0.19	0.36	0.36
	7	0.41	0.53	0.26	0.39	0.39
	8	0.4	0.55	0.25	0.42	0.42
	9	0.34	0.45	0.25	0.38	0.38
	10	0.36	0.5	0.25	0.41	0.41
	11	0.44	0.5	0.29	0.39	0.39
	12	0.4	0.5	0.29	0.35	0.35
	13	0.41	0.5	0.24	0.37	0.37
	14					
	15	NA	0.62	0.29	0.42	0.42
	16	0.38	0.48	0.24	0.37	0.37
	17					
	18	NA	0.52	0.32	0.42	0.42
	19	0.38	0.43	0.23	0.35	0.35
	20					
	21	0.42	0.48	0.23	0.35	0.35
	22	0.39	0.4	0.28	0.38	0.38
	23	0.4	0.55	0.25	0.39	0.39
	24	0.37	0.51	0.23	0.37	0.37
	25	0.49	0.53	0.27	0.45	0.45
	26					
	27	0.4	0.51	0.28	0.38	0.38
	28	0.36	0.47	0.21	0.37	0.37
CHD	1	0.4	0.48	0.27	0.42	0.42
	2	NA	0.45	0.29	0.41	0.41
	3	0.35	0.4	0.23	0.35	0.35
	4	0.38	0.43	0.24	0.33	0.33
	5	NA	0.62	0.28	0.37	0.37
	6	0.27	0.38	0.2	0.28	0.28
	7	0.46	0.48	0.28	0.4	0.4
	8	0.37	0.48	0.26	0.4	0.4
	9					
	10	0.37	0.49	0.25	0.37	0.37
	11	0.4	0.56	0.32	0.45	0.45
	12	0.38	0.44	0.21	0.33	0.33
	13	0.44	0.42	0.27	0.42	0.42
	14	0.47	0.57	0.27	0.37	0.37
	15	0.37	0.51	0.19	0.29	0.29
	16	0.35	0.45	0.26	0.34	0.34
	17	0.4	0.48	0.25	0.41	0.41
	18	0.33	0.45	0.22	0.36	0.36

19	0.31	0.52	0.28	0.4	0.4
20	0.38	0.48	0.26	0.39	0.39
21	NA	0.53	0.26	0.46	0.46
22					
23	0.39	0.5	0.24	0.33	0.33
24	0.39	0.52	0.31	0.4	0.4
25					
26	0.46	0.54	0.3	0.43	0.43
27	0.45	0.57	0.27	0.43	0.43
