# Supplementary material for: DNA methylation abnormalities of imprinted genes in congenital heart disease: a pilot study
Source: BMC Med Genomics. 2021 Jan 6;14:4. doi: 10.1186/s12920-020-00848-0 (PMC7789576; doi:10.1186/s12920-020-00848-0)
Supplement: Supplementary file 29 — Additional file 29: Table S20. CpG sites methylation level of 18 imprinted genes detected in CHD patients and healthy individuals. [file 12920_2020_848_MOESM29_ESM.pdf]

Table S20.1 CpG sites methylation level of SNRPN in CHD patients and healthy individuals

Groups	SampleID	CpG_1	CpG_2	CpG_3.4.5.6	CpG_7.8	CpG_9	CpG_10	CpG_11
Control	1	0.21	0.18	0.33	0.15	0.36	0.23	0.38
	2	0.26	0.27	0.29	0.11	0.29	0.27	0.38
	3							
	4	0.26	0.23	0.33	0.14	0.4	0.23	0.34
	5							
	6	0.27	0.3	0.39	0.16	0.29	0.33	0.4
	7	0.22	0.23	0.31	0.13	0.27	0.31	0.34
	8	0.23	0.34	0.44	0.21	0.4	0.4	0.43
	9	0.28	0.27	0.38	0.16	0.32	0.28	0.35
	10	0.26	0.2	0.36	0.15	0.39	0.28	0.38
	11	0.27	0.28	0.94	0.16	0.44	0.31	0.4
	12	0.21	0.27	0.36	0.14	0.26	0.28	0.39
	13	0.32	0.27	0.37	0.17	0.43	0.24	0.42
	14	0.22	0.24	0.32	0.11	0.32	0.25	0.33
	15	0.41	0.34	0.34	0.13	0.48	0.31	0.36
	16	0.26	0.22	0.31	0.13	0.36	0.26	0.35
	17	0.22	0.15	0.37	0.17	0.48	0.23	0.38
	18	0.24	0.24	0.37	0.17	0.42	0.32	0.37
	19	0.2	0.21	0.33	0.1	0.33	0.18	0.41
	20							
	21	0.23	0.19	0.32	0.12	0.3	0.25	0.33
	22	0.29	0.25	0.37	0.15	0.44	0.3	0.38
	23	0.29	0.22	0.37	0.16	0.37	0.31	0.37
	24	0.3	0.26	0.31	0.16	0.41	0.31	0.41
	25	0.36	0.4	0.48	0.2	0.35	0.4	0.41
	26	0.25	0.28	0.42	0.16	0.38	0.35	0.37
	27							
	28	0.32	0.34	0.34	0.19	0.43	0.33	0.31
CHD	1	0.22	0.2	0.35	0.16	0.26	0.26	0.31
	2	0.32	0.22	0.33	0.19	0.5	0.33	0.46
	3	0.4	0.25	0.39	0.18	0.32	0.3	0.38
	4	0.27	0.27	0.37	0.13	0.42	0.27	0.38
	5	0.25	0.2	0.36	0.15	0.38	0.26	0.35
	6	0.14	0.16	0.29	0.11	0.29	0.2	0.34
	7	0.29	0.23	0.84	0.15	0.39	0.25	1
	8	0.28	0.24	0.35	0.15	0.39	0.25	0.39
	9	0.25	0.22	0.36	0.14	0.31	0.31	0.34
	10	0.25	0.22	0.36	0.13	0.32	0.27	0.33
	11	0.31	0.21	0.37	0.19	0.43	0.32	0.39
	12	0.16	0.26	0.31	0.11	0.16	0.2	0.33
	13	0.25	0.21	0.32	0.16	0.36	0.34	0.34
	14							
	15	0.24	0.2	0.33	0.13	0.36	0.24	0.36
	16	0.26	0.13	0.33	0.14	0.4	0.28	0.38
	17	0.21	0.15	0.29	0.13	0.27	0.22	0.31

18	0.27	0.23	0.35	0.13	0.36	0.24	0.38
19	0.26	0.26	0.36	0.13	0.43	0.29	0.39
20	0.21	0.36	0.38	0.13	0.4	0.27	0.33
21	0.13	0.19	0.39	0.15	0.26	0.3	0.34
22	0.31	0.24	0.48	0.19	0.46	0.32	0.44
23	0.29	0.28	0.36	0.17	0.46	0.33	0.35
24	0.2	0.23	0.32	0.1	0.35	0.24	0.34
25							
26	0.14	0.21	0.35	0.11	0.25	0.26	0.34
27	0.25	0.22	0.38	0.16	0.36	0.28	0.35

Table S20.2 CpG sites methylation level of SNRPN in CHD patients and healthy individuals

Groups	SampleID	CpG_12	CpG_13.14.15	CpG_16	CpG_17	CpG_18
Control	1	0.37	0.47	0.32	0.19	0.47
	2	0.34	0.47	0.55	0.6	0.6
	3					
	4	0.35	0.48	0.36	0.28	0.53
	5					
	6	0.34	0.51	0.45	0.37	NA
	7	0.36	0.46	0.34	0.23	NA
	8	0.41	0.6	0.45	0.75	0.35
	9	0.34	0.54	0.39	0.43	0.28
	10	0.39	0.53	0.35	0.43	0.53
	11	0.33	0.54	0.31	0.41	NA
	12	0.36	0.47	0.34	0.19	0.46
	13	0.34	0.53	0.37	0.51	0.53
	14	0.32	0.44	0.26	0.29	NA
	15	0.34	0.49	0.42	0.48	0.61
	16	0.39	0.53	0.35	0.42	NA
	17	0.39	0.53	0.32	0.32	0.51
	18	0.4	0.51	0.38	0.47	0.59
	19	0.35	0.43	0.28	0.3	0.51
	20					
	21	0.36	0.49	0.39	0.3	0.39
	22	0.38	0.53	0.49	0.34	0.47
	23	0.37	0.52	0.33	0.38	0.29
	24	0.38	0.5	0.47	0.41	0.47
	25	0.42	0.55	0.4	0.47	0.62
	26	0.38	0.52	0.39	0.35	0.46
	27					
	28	0.39	0.56	0.38	0.39	0.44
CHD	1	0.36	0.52	0.32	0.3	0.2
	2	0.4	0.61	0.5	0.47	1
	3	0.41	0.56	0.45	0.44	0.53
	4	0.38	0.48	0.41	0.26	0.53
	5	0.33	0.5	0.35	0.41	0.42
	6	0.31	0.45	0.3	0.19	0.38
	7	0.36	0.51	0.39	0.37	0.97
	8	0.37	0.54	0.39	0.33	0.51
	9	0.36	0.47	0.38	0.36	0.46
	10	0.38	0.51	0.29	0.28	0.45
	11	0.41	0.59	0.38	0.37	0.46
	12	0.3	0.42	0.3	0.26	0.32
	13	0.37	0.51	0.43	0.42	0.4
	14					
	15	0.36	0.48	0.38	0.34	0.47
	16	0.36	0.5	0.35	0.31	0.48
	17	0.31	0.46	0.28	0.27	0.4

18	0.36	0.49	0.44	0.32	0.46
19	0.36	0.49	0.31	0.35	0.46
20	0.29	0.5	0.31	0.6	0.47
21	0.37	0.47	0.34	0.31	0.39
22	0.42	0.54	0.49	0.45	0.71
23	0.4	0.56	0.48	0.46	0.47
24	0.3	0.45	0.4	0.36	0.45
25					
26	0.3	0.37	0.22	0.19	0.33
27	0.34	0.49	0.38	0.27	0.38
