# Supplementary material for: DNA methylation abnormalities of imprinted genes in congenital heart disease: a pilot study
Source: BMC Med Genomics. 2021 Jan 6;14:4. doi: 10.1186/s12920-020-00848-0 (PMC7789576; doi:10.1186/s12920-020-00848-0)
Supplement: Supplementary file 30 — Additional file 30: Table S21. CpG sites methylation level of 18 imprinted genes detected in CHD patients and healthy individuals. [file 12920_2020_848_MOESM30_ESM.pdf]

Table S21 CpG sites methylation level of ZIM2 in CHD patients and healthy individuals

Groups	SampleID	CpG_1.2	CpG_3.4	CpG_5	CpG_6
Control	1	0.31	0.4	0.33	0.42
	2	0.33	0.24	0.38	0.32
	3	0.35	0.32	0.4	0.4
	4	0.31	0.22	0.36	0.35
	5	0.34	0.38	0.44	0.01
	6	0.35	0.27	0.41	0.4
	7	0.32	0.35	0.36	0.38
	8	0.33	0.38	0.42	0.47
	9				
	10	0.35	0.29	0.42	0.44
	11	0.42	0.36	0.5	0.45
	12	0.34	0.36	0.41	0.45
	13	0.34	0.3	0.4	0.43
	14	0.35	0.35	0.4	0.44
	15	0.35	0.41	0.38	0.39
	16	0.43	0.49	0.58	0.58
	17	0.33	0.34	0.42	0.42
	18	0.32	0.28	0.38	0.36
	19	0.35	0.32	0.43	0.43
	20				
	21	0.29	0.32	0.42	0.41
	22	0.33	0.3	0.4	0.39
	23	0.27	0.26	0.38	0.35
	24	0.36	0.34	0.4	0.45
	25	0.37	0.43	0.42	0.56
	26	0.32	0.27	0.39	0.37
	27	0.34	0.36	0.43	0.43
	28				
CHD	1	0.31	0.36	0.42	0.41
	2	0.29	0.24	0.35	0.34
	3	0.37	0.34	0.46	0.46
	4	0.28	0.15	0.31	0.27
	5	0.31	0.27	0.38	0.42
	6	0.27	0.24	0.37	0.33
	7				
	8	0.32	0.24	0.33	0.41
	9	0.28	0.3	0.4	0.39
	10	0.32	0.37	0.41	0.43
	11	0.37	0.38	0.39	0.44
	12	0.29	0.34	0.4	0.37
	13	0.35	0.45	0.42	0.38
	14	0.46	0.41	0.54	0.02
	15	0.34	0.37	0.38	0.55
	16	0.36	0.34	0.43	0.44
	17	0.41	0.46	0.49	0.55

18	0.34	0.29	0.43	0.42
19	0.32	0.3	0.39	0.41
20	0.4	0.32	0.44	0.47
21				
22	0.38	0.33	0.43	0.57
23				
24	0.34	0.26	0.4	0.34
25	0.33	0.33	0.44	0.47
26	0.46	0.36	0.5	0.47
27	0.29	0.26	0.36	0.35
